# JNK-Interacting Protein 3 Mediates the Retrograde Transport of Activated c-Jun N-Terminal Kinase and Lysosomes

**DOI:** 10.1371/journal.pgen.1003303

**Published:** 2013-02-28

**Authors:** Catherine M. Drerup, Alex V. Nechiporuk

**Affiliations:** Department of Cell and Developmental Biology, Oregon Health & Science University, Portland, Oregon, United States of America; Fred Hutchinson Cancer Research Center, United States of America

## Abstract

Retrograde axonal transport requires an intricate interaction between the dynein motor and its cargo. What mediates this interaction is largely unknown. Using forward genetics and a novel *in vivo* imaging approach, we identified JNK-interacting protein 3 (Jip3) as a direct mediator of dynein-based retrograde transport of activated (phosphorylated) c-Jun N-terminal Kinase (JNK) and lysosomes. Zebrafish *jip3* mutants (*jip3^nl7^*) displayed large axon terminal swellings that contained high levels of activated JNK and lysosomes, but not other retrograde cargos such as late endosomes and autophagosomes. Using *in vivo* analysis of axonal transport, we demonstrated that the terminal accumulations of activated JNK and lysosomes were due to a decreased frequency of retrograde movement of these cargos in *jip3^nl7^*, whereas anterograde transport was largely unaffected. Through rescue experiments with Jip3 engineered to lack the JNK binding domain and exogenous expression of constitutively active JNK, we further showed that loss of Jip3–JNK interaction underlies deficits in pJNK retrograde transport, which subsequently caused axon terminal swellings but not lysosome accumulation. Lysosome accumulation, rather, resulted from loss of lysosome association with dynein light intermediate chain (dynein accessory protein) in *jip3^nl7^*, as demonstrated by our co-transport analyses. Thus, our results demonstrate that Jip3 is necessary for the retrograde transport of two distinct cargos, active JNK and lysosomes. Furthermore, our data provide strong evidence that Jip3 in fact serves as an adapter protein linking these cargos to dynein.

## Author Summary

To form and maintain connections, neurons require the active transport of proteins and organelles between the neuronal cell body and axon terminals. Inhibition of this “axonal” transport has been linked to neurodegenerative diseases. Despite the importance of this process, to date there was no vertebrate model system where axonal transport could be studied in an intact animal. Our study introduces zebrafish as such a model and demonstrates its power for the analysis of axonal transport. We used this system to 1) initiate a genetic screen to find novel mediators of axonal transport; 2) develop *in vivo* imaging strategies to visualize axonal transport in real time in the intact animal; and 3) discover, using these methods, that JNK interacting protein 3 (Jip3) is required for the transport of two cargos, a kinase and lysosomes, from axon terminals to the cell body (retrograde transport). In the absence of Jip3, these cargos accumulate and axon terminals become dysmorphic, though the retrograde transport of other cargos is normal. Interestingly, abnormal localization of these cargos has been linked to axonal disease states, but our work is the first to identify a specific adapter protein necessary for their transport from axon terminals.

## Introduction

Active transport of proteins and organelles between the neuronal cell body and axon terminals is necessary for the formation and maintenance of functional neural circuits. Anterograde (to axon terminals) and retrograde (to the cell body) transport rely on motor proteins of the Kinesin and Dynein families respectively. These motors use the energy of ATP hydrolysis to walk along microtubule tracks, carrying cargo to its proper destination. Though 15 kinesin families exist in mammals [1], only 1 retrograde microtubule based motor protein, cytoplasmic dynein, is responsible for the majority of retrograde cargo transport in axons [2]–[4], leading to intriguing questions about the nature of dynein-cargo interaction specificity which have been largely unexplored [5].

The core cytoplasmic dynein motor is composed of an array of proteins that includes two motor domain-containing heavy chains, two intermediate chains, two light intermediate chains, and four light chains which bind the intermediate chains [6]. Though recombinant dynein heavy chain can function in microtubule sliding assays *in vitro* [7], dynein complex interacting proteins have been shown to be essential for the initiation of retrograde cargo movement *in vivo*. Dynactin, a large dynein-interacting protein complex, and Lis1 have been separately shown to be co-factors that are necessary for the initiation of retrograde transport [8]–[10]. Loss of either of these factors leads to decreased retrograde transport frequency of some cargo and can lead to the accumulation of dynein components as well as cargo in axon terminals [9]. Retrograde cargo is thought to either bind directly to the core dynein complex proteins or, alternatively, to additional adapter proteins. It is tempting to speculate that the use of distinct adapter proteins may confer specificity to motor-cargo interactions in the dynein motor system. Despite their importance for the understanding of dynein-based cargo transport, the identity of specific dynein cargo adapters is dramatically lacking [5].

We used the advantages of the zebrafish system, including its amenity to forward genetics and live imaging, to identify Jip3 (JNK-interacting protein 3) as a cargo-specific adapter for dynein-based axonal transport. Through a forward genetic screen, we isolated a mutant strain (*jip3^nl7^*) that exhibited swellings in axon terminals of long sensory axons, a potential sign of interrupted retrograde transport. *jip3^nl7^* carried a mutation in Jip3, a scaffold protein shown previously to serve as an adapter and facilitator of synaptic cargo anterograde transport through its interaction with Kinesin-1 [11]–[13]. In addition to anterograde transport machinery, Jip3 interacts with components of the dynein motor complex and c-Jun N-terminal Kinase (JNK). Indeed, Jip3 was first identified as a scaffold protein that links JNK to its upstream activating kinases, facilitating JNK activation [14]. Interestingly, Cavalli and colleagues demonstrated that Jip3 and activated JNK (pJNK) colocalized with p150^glued^ (dynactin complex protein) distal to sciatic nerve injury. Based on this data, they postulated that Jip3-JNK-dynein interaction may be important during retrograde damage signaling [15]. Furthermore, in this and other studies, Jip3 has been shown to biochemically interact with components of the retrograde motor complex, specifically p150^glued^ [15] and dynein light intermediate chain (DLIC; [13]). Thus, an intriguing possibility is that Jip3 could serve as an adapter for dynein-mediated retrograde transport of JNK and other cargo; however, neither this hypothesis nor the possibility that Jip3 is required for retrograde transport of any cargo, has been directly addressed to date.

Our work reveals discrete and direct roles for Jip3 in the retrograde transport of two cargos, pJNK and lysosomes. Using an *in vivo* imaging technique we developed for use in the zebrafish, we found specific retrograde transport defects in *jip3^nl7^*: frequencies of lysosome and pJNK retrograde transport were decreased causing accumulation of both cargos in axon terminals. Further analyses showed that direct Jip3-JNK interaction was necessary for retrograde clearance of pJNK from axon terminals and provided evidence that increased levels of pJNK were directly responsible for axon terminal swellings. Surprisingly, JNK activity and Jip3-JNK interaction had no impact on lysosome localization. Rather, co-transport analysis of lysosomes with both Jip3 and DLIC provided strong evidence that DLIC-lysosome interaction during retrograde transport relies on Jip3. Thus, based on our data we posit that Jip3 serves as an adapter protein for the retrograde transport of two distinct cargos, pJNK and lysosomes, and that failed retrograde clearance of pJNK contributes to the dysmorphic axon terminals in *jip3^nl7^* mutants.

## Results

### *jip3^nl7^* displays phenotypes consistent with impaired retrograde transport

**Figure 1:**
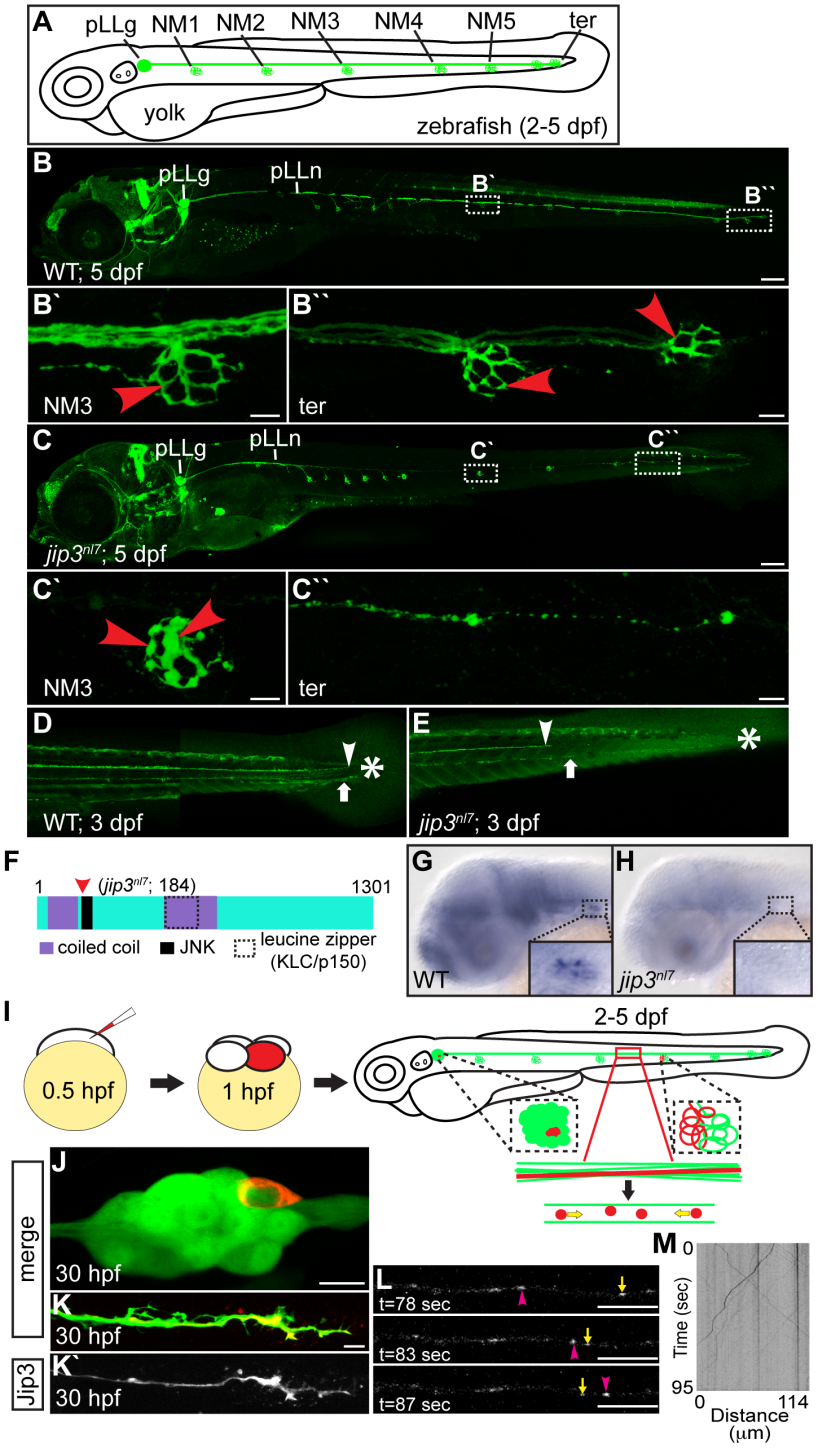
Jip3, an actively transported protein, was necessary for axon extension and the prevention of axon terminal swellings. (A) Schematic of a larval zebrafish illustrating the basic anatomy of the primary posterior lateral line (pLL) system. Neuromasts (NMs; terminal NM cluster-ter) are innervated by the pLL nerve (green), which emanates from the pLL ganglion (pLLg). (B) Wildtype *neurod:EGFP* transgenic at 5 dpf with the pLLg and pLL nerve (pLLn) indicated. (B′,B″) Panels illustrate pLL axon terminals that innervate NM3 (B′) and the distal end of the pLL nerve including the axon terminals at the terminal NM cluster (ter; B″; red arrowheads point to axon terminals). (C) *jip3^nl7^* mutants displayed truncated pLL nerves and distal pLL nerve thinning (C″) as well as swollen axon terminals in all NMs (NM3 shown in C′). Scale bars B and C = 100 µm. Scale bars in B′, B″, C′ and C″ = 10 µm. (D, E) Long central nervous system axons of the reticulospinal tract (arrowhead) and pLL efferent axons (arrow), visualized by the *phox2b:EGFP* transgenic reporter, were also truncated in *jip3^nl7^* mutants. End of trunk indicated by the asterisk. (F) Schematic of the zebrafish Jip3 protein showing conserved structural and binding domains. The red arrowhead indicates the location of the *jip3^nl7^* mutation, which generates a premature stop codon at amino acid 184. (G,H) *In situ* hybridization analysis revealed that *jip3* was expressed in the central and peripheral nervous systems at 2 dpf in wildtype but was lost in *jip3^nl7^*. (I) Schematic of the paradigm designed to image axon transport in the pLL nerve. (J) Transient expression of Jip3-mCherry in 1 neuron of the pLL ganglion at 30 hpf. (K) Jip3-mCherry was localized to a growth cone of an extending axon at 30 hpf. The pLL ganglion and nerve were visualized by expression of the *neurod:EGFP* transgene. (L) Jip3-mCherry is actively transported in pLL axons (Video S1). Arrowhead (pink) and arrow (yellow) indicate anterograde and retrograde particle movement respectively. (M) Kymograph of time-lapse imaging in J. Scale bars in J–L = 10 µm.

*jip3^nl7^* was isolated in a forward genetics screen for which we utilized the *TgBAC(neurod:EGFP)^nl1^* transgenic zebrafish (hereafter referred to as *neurod:EGFP*; [16]). This transgenic strain expresses an EGFP reporter in the central and peripheral nervous systems, including the posterior lateral line (pLL) ganglion and the long sensory axons emanating from it (Figure 1A, 1B; for screen details consult the Materials and Methods). We focused our screen on the long sensory axons of the pLL because of their planar character and superficial localization. These axons originate from the pLL ganglion, located just posterior to the ear, and extend along the trunk, branching to innervate mechanosensory hair cells that reside within surface sensory organs called neuromasts (NMs; axon terminals innervating NM3 and terminal NMs are shown in Figure 1B′ and 1B″ respectively). Initial pLL nerve extension and NM formation is complete by 2 dpf (days post-fertilization), and by 5 dpf a functional neural circuit has developed between NM hair cells and afferent pLL axons [17]. The recessive *jip3^nl7^* mutant (Figure 1C) was isolated because it displayed truncation of pLL axons (incomplete penetrance; Figure 1C″) and swollen axon terminals innervating all trunk NMs (penetrance 100%; NM3 in Figure 1C′ and data not shown). To determine if long central nervous system axons were also affected by loss of Jip3, we analyzed axons of the reticulospinal tract as well as the efferent axons that project from the CNS to innervate the pLL NMs by crossing the *jip3^nl7^* mutation into the *TgBAC*(*phox2b:EGFP*)*^w37^* transgenic line [18]. Similar to pLL afferents, both reticulospinal tract and pLL efferent axons were truncated in *jip3^nl7^* mutants (Figure 1D, 1E). *jip3^nl7^* mutants were homozygous viable and the pLL axonal phenotype did not have a maternal component, as progeny derived from homozygous crosses displayed identical phenotypes to that of progeny derived from heterozygous crosses (data not shown).

We used a positional cloning approach to isolate the genomic locus containing the *jip3^nl7^* gene mutation. Zebrafish Jip3, which mapped to this locus, is similar to its mammalian orthologs and contains two coiled coil domains, one leucine zipper deemed integral for Kinesin Light Chain (KLC) and dynactin binding [19], [20], and a JNK binding domain (Figure 1F). Sequencing of *jip3* from *jip3^nl7^* mutants revealed a mutation at nucleotide 552 which created a premature stop codon, truncating the Jip3 protein at amino acid 184 (Figure 1F). *In situ* hybridization analysis showed that, similar to mouse [21], *jip3* was expressed in the central and peripheral nervous systems of the zebrafish embryo (Figure 1G). *jip3* expression was lost in *jip3^nl7^*, perhaps due to nonsense-mediated mRNA decay (Figure 1H). Consequently, *jip3^nl7^* is likely a Jip3 null. Initial investigations revealed the pLL nerve phenotypes were not due to impaired pLL patterning, neuronal cell death, abnormal glial support/myelination, or gross cytoskeletal abnormalities (Figure S1). As Jip3 has been shown to interact with members of the anterograde and retrograde motor complexes [11]–[13], [22], [23] and interruptions in transport have been associated with axon swellings like those observed in *jip3^nl7^* [24], [25], we next focused our investigations on the potential function of Jip3 in axonal transport.

### *In vivo* analysis of Jip3 transport in the zebrafish pLL nerve

To study the function of Jip3 in axonal transport, we developed methods to visualize microtubule-based axonal transport in the pLL system *in vivo*, in intact zebrafish embryos and larvae (Figure 1I). Zebrafish are ideal for such a preparation as they are transparent through early embryonic and larval development, facilitating *in vivo* live imaging, and transient transgenesis can be used reliably to express tagged cargos of interest mosaically. Using these advantages, we developed a protocol that requires no surgical or invasive techniques to visualize protein or organelle transport in the long and planar axons of the pLL. To image axonal transport in zebrafish pLL axons, zygotes are injected with DNA encoding a cargo of interest tagged with a fluorescent reporter. Expression of these constructs is controlled by a neuron-specific 5 kilobase portion of the *neurod* promoter (*5kbneurod*; [26]). This results in mosaic expression of the desired cargo in the pLL ganglion, which, in ideal preparations, labels 1 to 2 neurons. Neurons expressing cargo are then monitored for full axon extension, innervation of NMs, and the absence of cargo accumulation in neuronal cell bodies and axons to assess optimal concentrations of DNA for injection. Using this approach, cargo transport can be visualized in individual pLL axons during axon extension (1–2 dpf), post-extension (after 2 dpf), and after functional synaptic connections are established (5 dpf).

We first utilized this technique to observe the localization and transport of a Jip3-mCherry fusion in pLL neurons and their axons. During axon extension (30 hpf), Jip3-mCherry localized to the neuronal cell body and axon growth cones (Figure 1J, 1K), similar to Jip3 localization in cultured neurons [27]. We then visualized Jip3 transport at 2 dpf, just after pLL nerve extension completes, and analyzed transport parameters using kymograph analysis (Figure 1L, 1M and Video S1). Jip3-containing cargo traveled at average velocities of 1.60 µm/sec in the anterograde direction and 1.35 µm/sec when moving in the retrograde direction (N = 7 larvae); these parameters are consistent with fast anterograde and retrograde transport [1].

### Defects in organelle transport in *jip3^nl7^* mutants

Next, we assayed the localization and transport of ssNPY-mCherry [28], a marker of Golgi-derived vesicles, to determine if loss of Jip3 affects the axonal transport of this generalized cargo. At 5 dpf, we observed large accumulations of mCherry positive puncta in axon terminals of *jip3^nl7^* mutants but not in wildtype siblings (Figure S2A, S2B; for this and other experiments, mutants were identified using the genotyping protocol described in the Materials and Methods, except where otherwise indicated). *In vivo* imaging and kymograph analysis demonstrated bidirectional movement of mCherry-positive puncta in wildtype and *jip3^nl7^* mutants (Figure S2C–S2F; Videos S2 and S3) with decreased frequency of anterograde and retrograde transport of this cargo in *jip3^nl7^* at 2 dpf with a tendency toward a decrease at 5 dpf (Figure S2G). Neither distance nor velocity of cargo movement were altered (Figure S2H, S2I), potentially implicating Jip3 in cargo-motor attachment, rather than modulation of motor activity.

**Figure 2:**
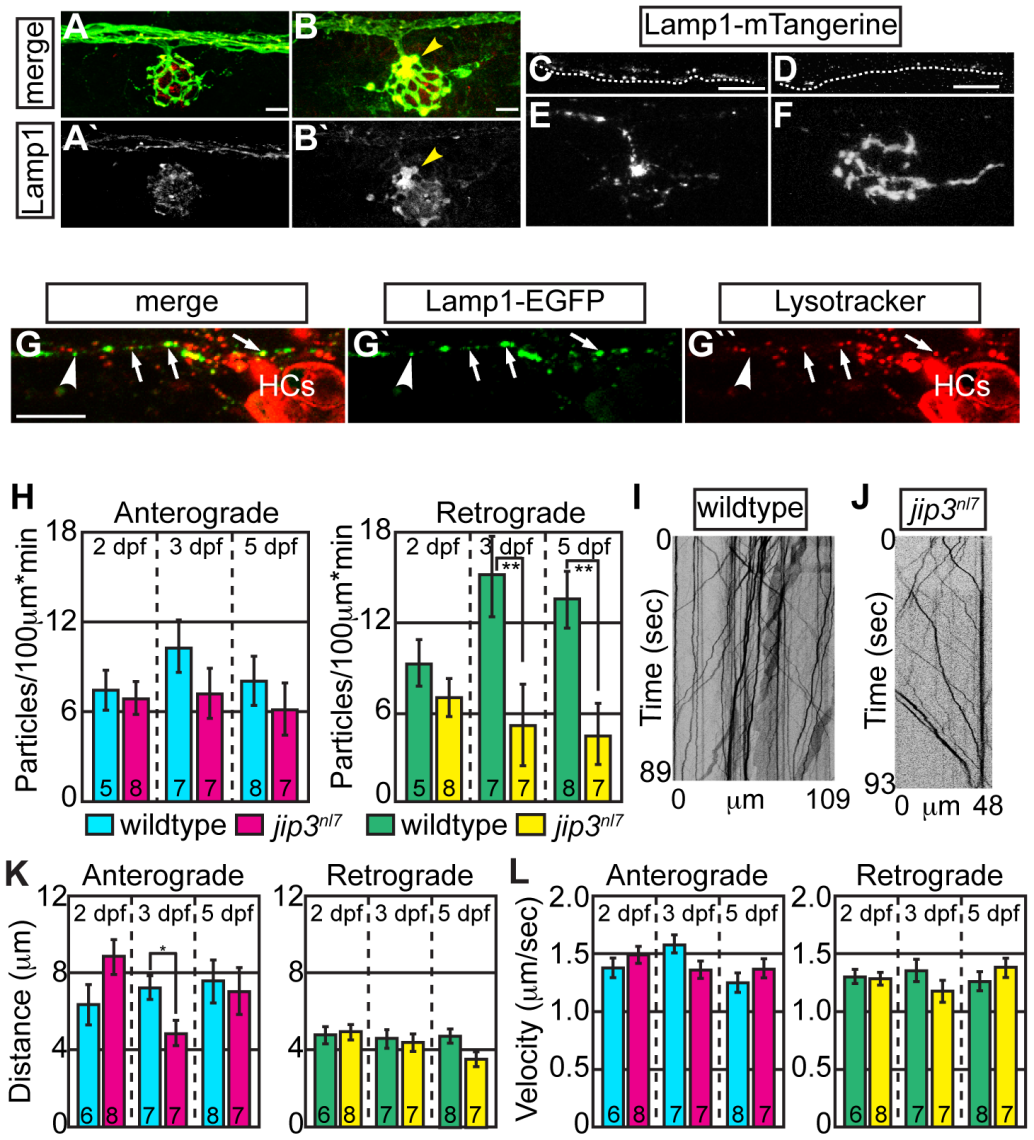
Lysosomes accumulated in *jip3^nl7^* axon terminal swellings. (A,B) Lysosome density, as assayed by Lamp1 immunolabeling (A,B-red, A′B′-white) was increased in *jip3^nl7^* NM3 axon terminals at 5 dpf (arrowhead). Larvae carried the *neurod:EGFP* transgene to label pLL axons. (C,D) Stills from imaging sessions of Lamp1-mTangerine transport in the pLL nerve of wildtype (C) and *jip3^nl7^* mutant (D) embryos at 3 dpf (Videos S4 and S5). Dotted lines denote the lower bound of the axons imaged. (E,F) Lamp1-mTangerine accumulated in the axon terminals of *jip3^nl7^* mutants (F) but not wildtype siblings (E) at 3 dpf (NM1 shown for both). (G) The majority of Lamp1-EGFP positive vesicles in axons co-labeled with Lysotracker red, indicating they were lysosomes. Arrows denote a subset of the Lamp1-EGFP/Lysotracker red co-labeled vesicles. Arrowhead points to a small Lamp1 positive vesicle that was not acidified. HC denotes hair cells rich in Lysotracker red positive, acidic vesicles. (H) Retrograde, but not anterograde, lysosome transport frequency was decreased in *jip3^nl7^* mutants at 3 and 5 dpf (Wilcoxon rank-sum; **-*p*<0.005). Number of embryos analyzed is indicated on the graph for this and all subsequent bar graphs. (I,J) Kymographs of wildtype (I) and *jip3^nl7^* (J) Lamp1-mTangerine transport shown in C and D. (K,L) Neither distance moved in individual bouts (K) nor velocity of movement (L) were altered in *jip3^nl7^* mutants, save a decrease in anterograde transport distance at 3 dpf (Wilcoxon rank-sum; *p<0.05). Scale bars = 10 µm.

Next, we set out to determine the identity of the mCherry labeled retrograde cargo(s) by looking for accumulation of commonly transported retrograde cargos in *jip3^nl7^* axon terminals using immunofluorescence [29], [30]. Neither late endosomes (Rab7-positive) nor autophagosomes (LC3-positive) accumulated in *jip3^nl7^* axon terminals (Figure S3A–S3D). Consistent with a previous study on Jip3's role in anterograde transport of TrkB [13], TrkB levels were decreased in *jip3^nl7^* axon terminals, as assayed by TrkB antibody labeling (Figure S3E, S3F). In contrast, the axon terminal swellings in *jip3^nl7^* were rich in lysosomes that were visualized using two separate markers, Lamp1 (detected by immunofluorescence; Figure 2A, 2B) and Lysotracker red (vital dye; Figure S3G, S3H).

We then asked whether abnormalities in lysosomal transport caused lysosome accumulations in axon terminals by employing our *in vivo* imaging approach, using a Lamp1-mTangerine fusion [31] to mark lysosomes in pLL axons (Figure 2C–2F; see Videos S4 and S5). The ability of a Lamp1-EGFP fusion construct to label lysosomes was confirmed by double labeling with the vital dye Lysotracker red (Figure 2G). Similar to our immunolabeling results, Lamp1-mTangerine accumulated in the axon terminals of *jip3^nl7^* mutants but not wildtype controls (Figure 2E, 2F). Live imaging analysis demonstrated that, though Lamp1-mTangerine transport parameters were not altered at 2 dpf, the number of lysosomes moving in the retrograde direction was significantly decreased at 3 dpf in *jip3^nl7^* axons (Figure 2H–2J; WT = 15.08±2.71 vs. *jip3^nl7^* = 5.14±2.71 particles/100 µm*min, *p*≤0.002; Wilcoxon rank-sum). A similarly reduced frequency of lysosome retrograde transport was also observed at 5 dpf, while distance and velocity of movement were largely unaffected at all stages (Figure 2K, 2L). These data show that retrograde lysosome transport relies on Jip3.

### Jip3 is necessary for retrograde pJNK transport

**Figure 3:**
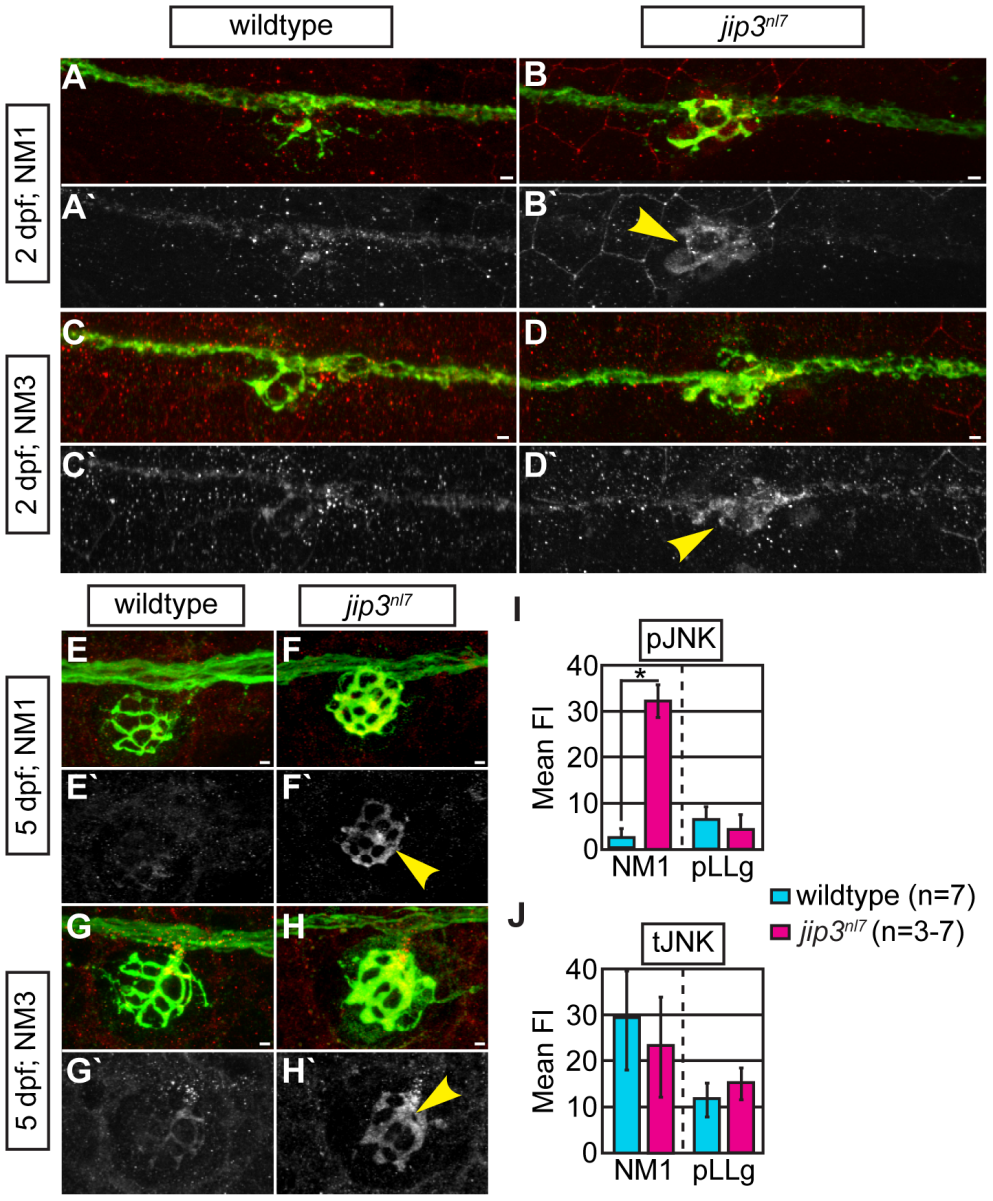
pJNK levels were elevated in *jip3^nl7^* axon terminals. (A–H) Immunolabeling for active JNK (pJNK; red in merge; white in single channel) in proximal (NM1) and distal (NM3) neuromasts at 2 and 5 dpf. pJnk levels were elevated in all axon terminals in *jip3^nl7^* mutants (A–I; arrowheads). (I,J) Mean fluorescent intensity (background subtracted; see Materials and Methods for details) of pJNK and total JNK (tJNK) labeling in NM1 axon terminals and the pLL ganglion (pLLg) at 5 dpf. (ANOVA, post-hoc contrasts; *-*p*<0.001). Scale bars = 10 µm.

Jip3 has been shown to interact with components of the Kinesin-1 motor to regulate anterograde transport [11]–[13], but a role for Jip3 in retrograde transport has not been described previously. Therefore, we next sought to address how Jip3 functioned to regulate retrograde axonal transport. Jip3 was originally identified as a JNK-interacting protein and has been shown to facilitate JNK activation *in vitro* [14]. Thus, we would predict that loss of Jip3 would lead to decreased JNK activation. As JNK activity can impact numerous intracellular processes that could potentially affect axonal transport machinery [32], [33], we assayed levels and localization of active JNK (pJNK) using pan-pJNK immunolabeling. Surprisingly, instead of a decrease, we found *elevated* levels of pJNK in the mutant axon terminals innervating all NMs from 2 dpf onward (Figure 3A–3I and data not shown; see Materials and Methods for an explanation of fluorescent intensity measurement). In contrast, total JNK (tJNK) levels in *jip3^nl7^* were comparable to controls (Figure 3J and Figure S4A–S4D). Western blot analysis of whole embryo extracts revealed no increase in overall tJNK or pJNK levels in *jip3^nl7^* (Figure S4E, S4F), pointing to a change in localization of pJNK rather than overall JNK expression or activity.

**Figure 4:**
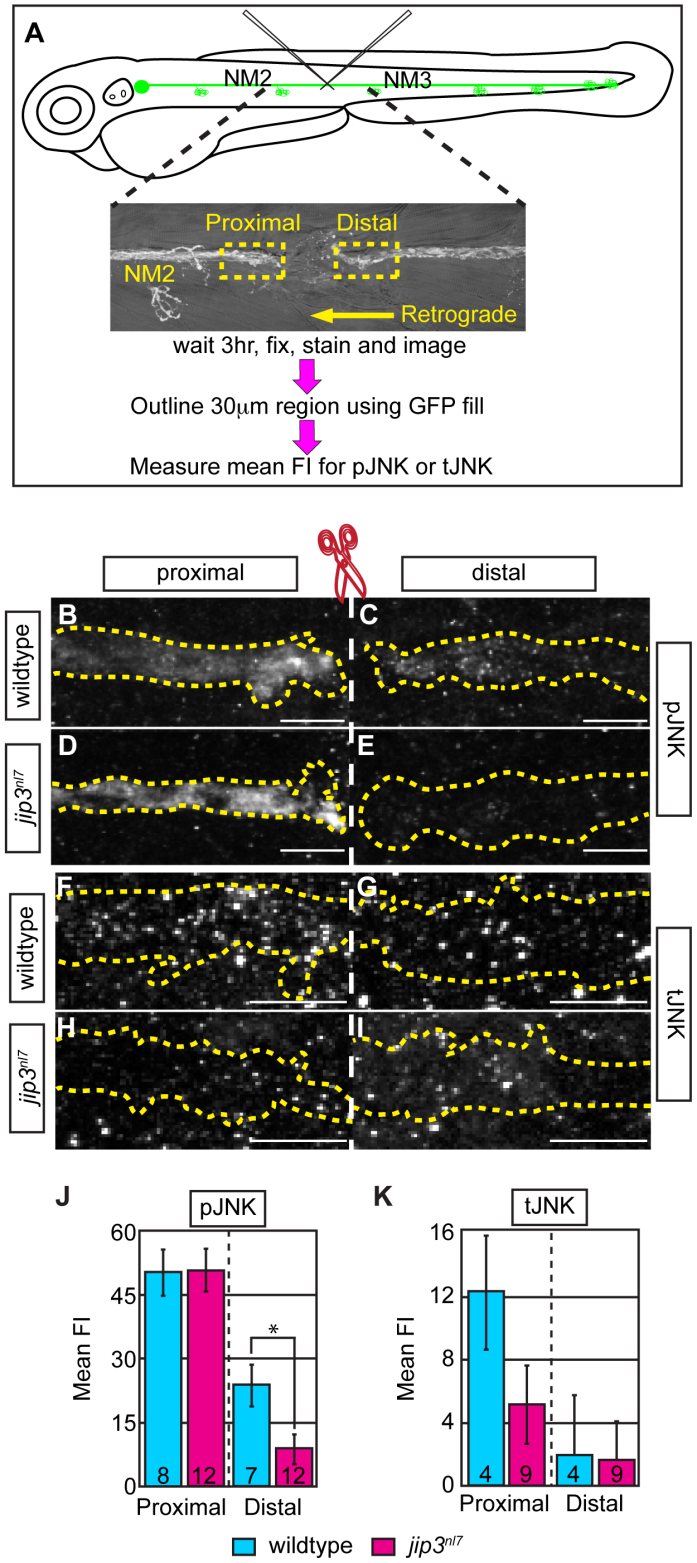
pJNK failed to accumulate distal to injury. (A) Schematic and time-line of the injury model experiment and fluorescent intensity quantification. pLL nerve (identified using the *neurod:EGFP* transgene) was severed using finely pulled glass capillaries. DIC image of a representative injury illustrates peripheral tissue remained mostly intact. Three hours post-injury, larvae were fixed and stained for GFP (to identify the nerve) and either pJNK or tJNK. Thirty µm areas immediately proximal or distal to the injury were imaged and the mean fluorescent intensity of pJNK or tJNK was determined in summed projected stacks through the nerve only in areas that overlapped with GFP expression (outlined by dotted lines in B–I). Background mean fluorescent intensity was determined in adjacent tissue. (B–I) Proximal and distal nerve (dotted outline) adjacent to site of injury (dashed line) in wildtype and *jip3^nl7^* larvae immunolabled for pJNK (B–E) and tJNK (F–I). (J) Levels of pJNK were decreased distal to nerve injury in *jip3^nl7^* but proximal levels were comparable to wildtype (ANOVA, post-hoc contrasts; *-*p*<0.05). (K) tJNK levels trended towards a decrease proximal to the site of injury in *jip3^nl7^* (ANOVA, post-hoc contrasts; *p*<0.1790) but were not different in the retrograde pool, distal to axonal severing.

Given the ability of Jip3 to bind components of the retrograde motor and pJNK [14], [15], we reasoned that Jip3 might directly mediate pJNK retrograde transport/clearance from axon terminals by attaching this active kinase to the dynein motor complex. To determine if Jip3 has a specific role in pJNK transport, we used two complimentary approaches. First, we developed an axon injury model for use in the zebrafish pLL nerve to indirectly assay pJNK transport, similar to a protocol previously used in mouse sciatic nerve (Figure 4A; see Materials and Methods for procedure details; [15]). Following injury, cargos that are transported in the anterograde direction will accumulate proximal to the injury site, whereas retrograde cargos will accumulate distal to the injury site. Severing the pLL nerve between NM2 and NM3 at 5 dpf resulted in accumulation of pJNK in the pLL nerve proximal and distal to the site of injury in wildtype larvae by 3 hours post-injury. In contrast, pJNK failed to accumulate distal to the site of injury in *jip3^nl7^* mutants (Figure 4B–4E, 4J), indicating failed retrograde pJNK transport in mutant axons. Total JNK levels were not significantly different proximal or distal to injury site in *jip3^nl7^* mutants (Figure 4F–4I, 4K), though there was a strong trend towards decreased levels of the tJNK anterograde pool (proximal to the injury site) in *jip3^nl7^* mutants. This data supports the hypothesis that loss of Jip3 inhibits pJNK retrograde transport, which would lead to accumulations of this kinase in axon terminals.

Next, we asked whether dynein motor components were normally transported to axon terminals in *jip3^nl7^* mutants, as the perturbation of this transport could indirectly affect retrograde cargo movement. Using immunolabeling for two components of the dynein complex (Dynein heavy chain and p150^glued^), we demonstrated proper localization of these core dynein motor proteins to *jip3^nl7^* mutants, confirming that the retrograde motor can reach axon terminals in *jip3^nl7^* mutants (Figure S5A–S5G). From this data, we can also infer that even in the absence of Jip3, the initiation of dynactin-mediated, dynein movement was intact since these retrograde motor components did not accumulate in axon terminals [9], [10].

**Figure 5:**
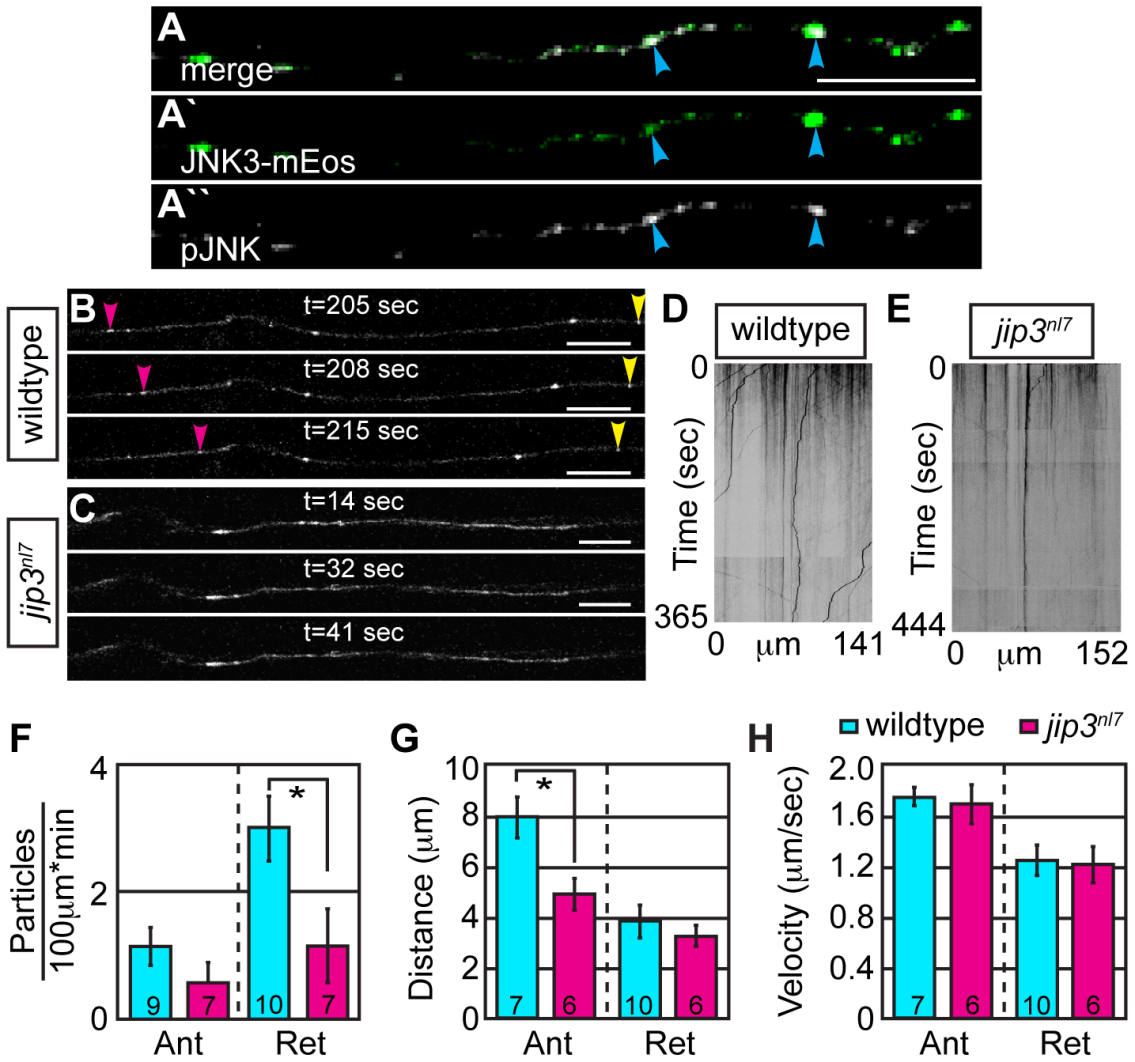
Retrograde JNK3 transport frequency was decreased in *jip3^nl7^* mutants. (A) Immunolabeling for pJNK in an axon expressing JNK3-mEos showed a high degree of colocalization (arrowheads) indicating that a large percentage of axonal JNK3-mEos is activated. (B,C) Representative stills from a live imaging session showing axonal transport of JNK3-mEos in a pLL axon of a wildtype (B) and *jip3^nl7^* mutant (C) at 2 dpf (see Videos S6 and S7). Pink arrowhead denotes anterograde movement, yellow retrograde movement. (D,E) Kymographs generated from these imaging sessions. (F) Number of retrograde JNK3-mEos puncta (corrected for size of analyzed region and time of imaging session) was decreased in *jip3^nl7^* (ANOVA, post-hoc contrasts; *-*p*<0.05). Distance of individual retrograde movement bouts (G) and velocity (H) were unaltered in *jip3^nl7^*. Anterograde transport distance was decreased (*-*p*<0.05; Ant = anterograde; Ret = retrograde). Scale bars = 10 µm.

Finally, we used our *in vivo* live imaging to concretely determine if retrograde JNK transport was impaired in *jip3^nl7^* mutant pLL axons using transient expression of JNK3 tagged with mEos. We chose to use JNK3 for our *in vivo* analysis because Jip3 has been shown to bind most strongly to the JNK3 homolog [14], and *jnk3* is strongly expressed in the zebrafish nervous system (Figure S6A, S6B). Phospho-JNK immunolabeling of embryos expressing JNK3-mEos driven by the *5kbneurod* promoter in pLL axons demonstrated that a large portion of JNK3-mEos positive vesicles carried the active form of this kinase (Figure 5A). Live imaging experiments revealed JNK3-mEos positive puncta traveled bidirectionally in wildtype and *jip3^nl7^* mutants at 2 dpf (Figure 5B, 5C; Videos S6 and S7). Using kymograph analysis (Figure 5D, 5E), we found a decrease in the number of JNK3-mEos positive puncta moving in the retrograde direction at 2 dpf in *jip3^nl7^* mutants (Figure 5F; wildtype:2.99±0.48 vs. *jip3^nl7^*:1.15±0.58 particles/100 µm*min, *p*≤0.05; Wilcoxon rank-sum) while retrograde movement distance and velocity were largely unchanged (Figure 5G, 5H). Taken together with the results from our injury model, these data confirmed that the frequency of retrograde pJNK transport was hindered in *jip3^nl7^* mutants.

### Jip3-JNK interaction is necessary for pJNK retrograde transport

Based on our data and previous work showing that Jip3 can bind components of the dynein motor complex [15], we hypothesized that direct Jip3-JNK interaction was necessary for the retrograde transport of pJNK. To address this, we first asked whether Jip3 and JNK3 were transported together in pLL axons using a dual cargo transport assay. We co-injected Jip3-mCherry and JNK3-mEos plasmids and identified embryos in which both constructs were expressed in the same pLL neuron. Notably, co-injection of these and other cargos used for dual transport analysis (see below) resulted in almost 100% co-expression. Sequential imaging of Jip3 and JNK3 positive vesicles at 2 dpf revealed a high degree of co-transport, primarily in the retrograde direction (Video S8). While only 16% of vesicles in the anterograde pool were positive for both Jip3 and JNK3, 87% of vesicles in the retrograde pool carried both proteins (N = 5 embryos). This data supported a role for Jip3 in the retrograde transport of activated JNK. Importantly, since mEos is a green to red photoconvertable molecule, we used extreme caution during these dual imaging experiments to prevent accidental photoconversion and noted no green to red shift in the vesicles imaged during these sessions (data not shown).

**Figure 6:**
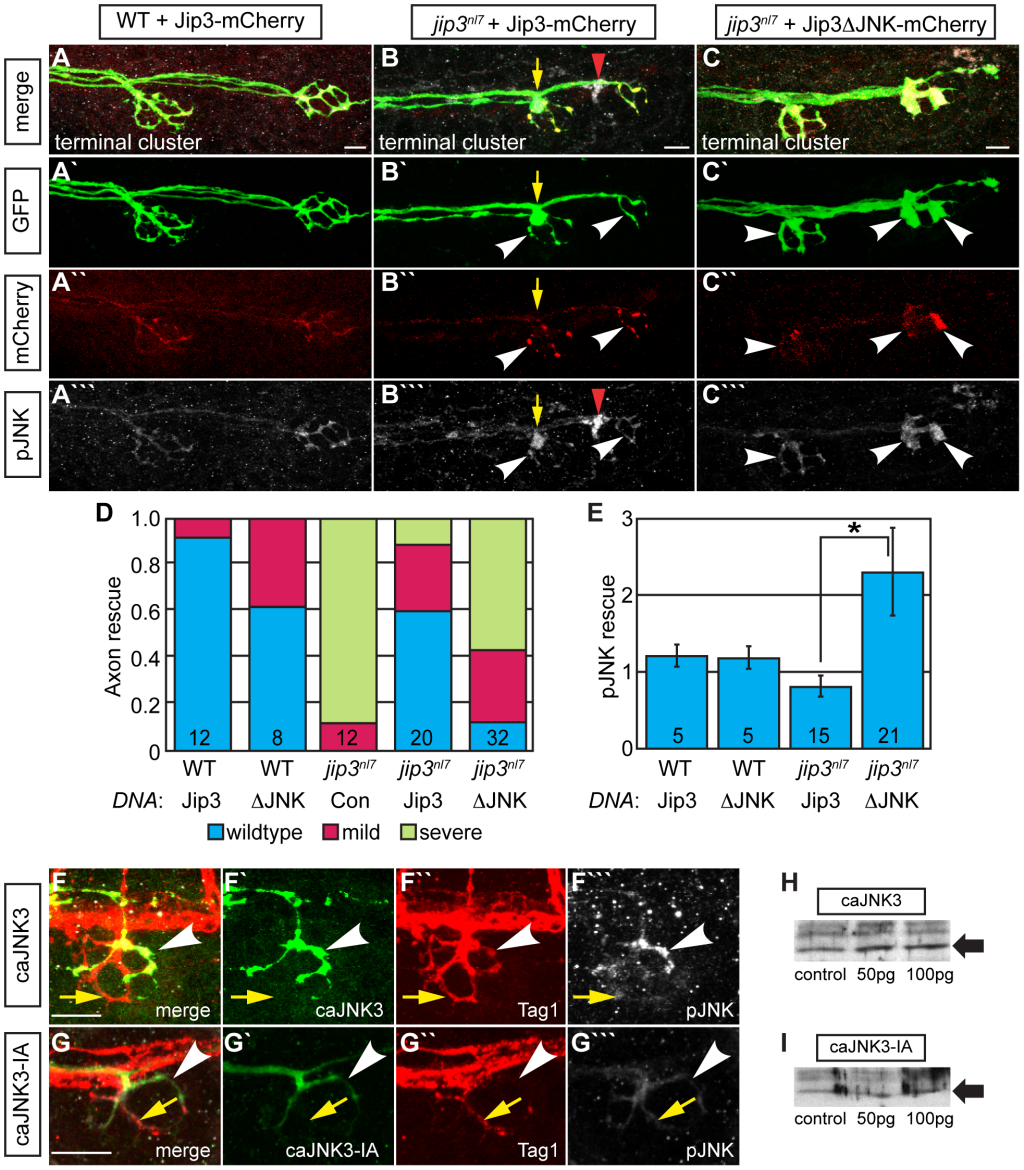
Jip3 interaction with JNK was necessary for pJNK clearance and the prevention of axon swellings. (A–C) Axon terminal swellings and pJNK accumulation were rescued by Jip3 but not by Jip3ΔJNK. Axonal swellings were visualized live by *neurod:EGFP* transgene expression; following live imaging, pJNK was assayed by individual larva immunolabeling at 4 dpf. White arrowheads mark axon terminals expressing the DNA constructs. Yellow arrow points to a swelling in an axon not expressing Jip3-mCherry. Red arrowhead denotes an underlying pJNK positive cell not expressing Jip3-mCherry. (D) Ratio of axon terminal swellings in each class (mild = small swellings, severe = large swellings) show rescue of axonal swellings by full-length Jip3 but not Jip3ΔJNK. *DNA* indicates the rescue construct injected; Jip3 = full-length Jip3-mCherry; ΔJNK = Jip3ΔJNK-mCherry; Con = uninjected control. Embryo genotype, determined by control axon terminal morphology, is indicated below each bar. (E) Ratio of pJNK levels in Jip3 or Jip3ΔJNK expressing axon terminals to those not expressing the rescue construct at 4 dpf. Jip3, but not Jip3ΔJNK, suppressed increased pJNK levels in *jip3^nl7^* (Wilcoxon rank-sum; *-*p*<0.01). (F) Induction of constitutively active JNK3 tagged with EGFP (caJNK3; green) for 15 hours at 4 dpf increased the level of pJNK immunofluorescence concomitant with the induction of swellings shown by both the caJNK3-EGFP fill and Tag1 immunolabeling of neuronal membranes. Arrowhead points to a caJNK3-EGFP expressing axon. Yellow arrow indicates an axon terminal in the same NM not expressing this construct. (G) Axon terminal swellings were absent in axon terminals expressing an inactive form of the same construct (caJNK3-IA), indicating that JNK activation was necessary to induce swellings. (H,I) Efficacy of both caJNK3 and caJNK3-IA were assayed by Western blot analysis of phospho-cJun, a downstream target of active JNK. While whole embryo overexpression of caJNK3-EGFP by RNA injection induced elevated levels of phospho-cJun at 24 hpf (H), similar expression of the inactive form of caJNK3-EGFP (caJNK3-IA) failed to induce a similar increase (I). Scale bars = 10 µm.

Next, we addressed whether the direct interaction between Jip3 and JNK was necessary for retrograde pJNK transport by asking whether the pJNK accumulation in *jip3^nl7^* could be rescued with a Jip3 variant that lacked the JNK binding domain (Jip3ΔJNK: amino acids 202–214; [32]). DNA constructs were injected into zygotes to mosaically express Jip3-mCherry or Jip3ΔJNK-mCherry in individual pLL ganglion neurons. At 4 dpf, axon terminals expressing the respective fusions were imaged live and scored for axon morphology before larvae were individually immunolabeled for pJNK and the same axon terminals were re-imaged. As each NM is innervated by 2 axons and this innervation is segregated in space [34], we could use the non-expressing half of the NM to identify which larvae were *jip3^nl7^* mutants as well as utilize it as a normalizing factor for the quantification of pJNK immunofluorescence. Though full-length Jip3 rescued axon terminal swellings and the accumulation of pJNK, Jip3ΔJNK was unable to rescue either phenotype (Figure 6A–6E). Importantly, expression of Jip3ΔJNK by mRNA injection rescued axon length, providing evidence that deletion of this region did not result in protein instability or failed processing, and pointing to a JNK-independent mechanism for Jip3's role in axon outgrowth (Figure S7). In summary, these data show that direct interaction between Jip3 and JNK is necessary for pJNK retrograde transport and also revealed a correlation between the accumulation of pJNK due to loss of Jip3-JNK interaction and the generation of axon terminal swellings.

### Elevated pJNK is sufficient to induce axon terminal swellings

To determine if high levels of pJNK in axon terminals were sufficient to cause axon terminal swellings, we conditionally and mosaically expressed a constitutively active form of JNK3 (caJNK3; [35], [36]) fused to EGFP under the control of a heat shock promoter in pLL neurons of wildtype larvae. Fifteen hours after activation at 4 dpf, we identified larvae that were expressing this construct in pLL axon terminals. Subsequently, these larvae were individually immunolabeled using anti-pJNK and anti-GFP antibodies to determine if caJNK3 could alter axonal morphology and additionally determine if axonal swellings correlated with elevated pJNK levels. Using this assay, we found that increased pJNK levels by expression of caJNK3 correlated with the presence of axon terminal swellings (Figure 6F). Interestingly, expression of caJNK3 did not always elevate pJNK levels (8 out of 17 larvae) and axon terminals were not swollen in these instances (data not shown). To test if axon terminal swellings were a result of JNK activity, we mutated the site phosphorylated by the upstream activating MAPKK to render caJNK3 inactive (caJNK3-IA; [37]). To assay the efficacy of the caJNK3 and caJNK3-IA constructs, we expressed both individually using RNA-mediated whole embryo expression and assayed phospho-cJun levels, a direct downstream JNK target, by Western blot analysis. As predicted, caJNK3 elevated levels of p-cJun (Figure 6H) while caJNK3-IA did not (Figure 6I). Induction of caJNK3-IA using a protocol identical to that used of caJNK3 did not cause axonal swellings in any of the 16 larvae we imaged (Figure 6G), confirming that JNK activity was indeed required for the generation of axon terminal swellings. These experiments demonstrated that high JNK activity is sufficient to induce axonal swellings and provided strong evidence that the axon terminal swellings in *jip3^nl7^* mutants are due to increased pJNK levels at axon terminals.

### Lysosome accumulation is independent of pJNK levels and Jip3-JNK interaction

**Figure 7:**
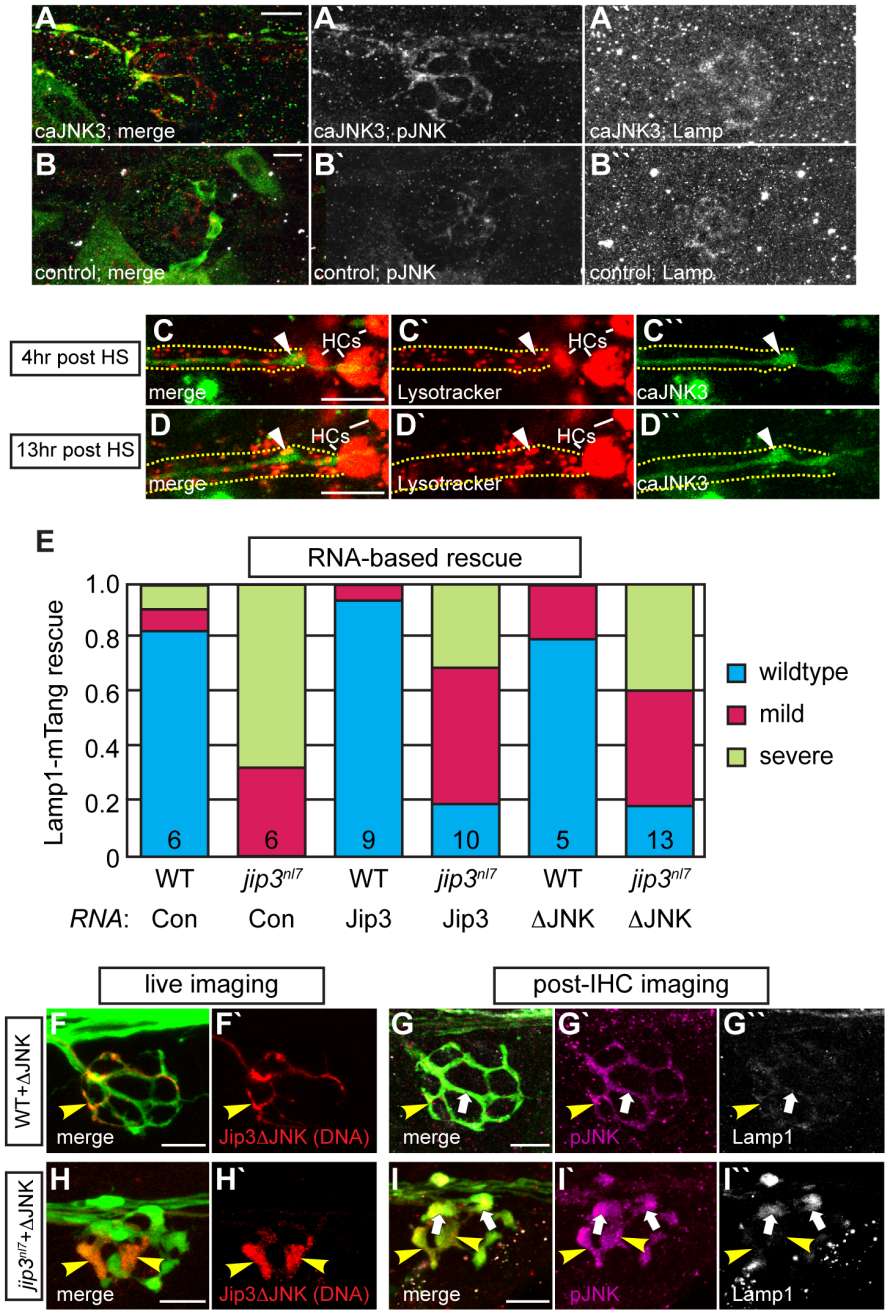
Increased levels of pJNK did not cause lysosome accumulation in *jip3^nl7^*. (A) Induction of caJNK3-EGFP at 4 dpf increased the level of pJNK immunofluorescence (middle) in a subset of axon terminals but did not lead to lysosome accumulation as compared to control (B). Scale bars = 10 µm. (C, D) This result was confirmed by Lysotracker red labeling. Surrounding, non-caJNK3-EGFP positive axons show similar numbers, size and density of lysosomes both 4 hours and 13 hours after induction of caJNK3. The pLL nerve was visualized by phase contrast optics and is outlined. Arrowhead indicates axonal swellings caused by high levels of activated JNK. HC denotes neuromast hair cells that strongly label with Lysotracker red. (E) Whole embryo expression of Jip3 and Jip3ΔJNK by mRNA injection partially suppressed the accumulation of lysosomes in *jip3^nl7^* mutant axon terminals at 3 dpf as assayed by expression of Lamp1-mTangerine in pLL neurons. Wildtype – Lamp1-mTangerine positive small puncta only; Mild – small puncta and aggregates visible; Severe - few to no small puncta apparent and large aggregations of Lamp1-mTangerine. (F–I) Injection of 10 pg of a DNA construct encoding Jip3ΔJNK-mCherry rescued lysosome accumulation in *jip3^nl7^* axon terminals. Larvae that expressed Jip3ΔJNK-mCherry (red) in pLL axons and carried the *neurod:EGFP* transgene were first imaged live (F,H) to identify expressing axon terminals. They were then individually fixed, stained for pJNK (pseudo-colored magenta) and Lamp1 (white), and subsequently the same axon terminals were reimaged (G,I). Arrowheads point to axon terminals in wildtype (F,G; NM1) and *jip3^nl7^* (H,I; NM5) that express Jip3ΔJNK-mCherry (red) at 5 dpf. Arrows point to axon terminals in the same NMs that did not express this construct. Note that expression of Jip3ΔJNK-mCherry in *jip3^nl7^* completely rescued lysosome accumulation (yellow arrowheads in I″) but failed to rescue high levels of pJNK (yellow arrowheads in I′).

Our data demonstrated that lysosomes accumulate in *jip3^nl7^* mutant axon terminals (see Figure 2) and elevated pJNK levels cause axon terminal swellings (see Figure 6). Next, we asked whether elevated pJNK could cause lysosomal accumulation. To test this, we used the approach described above to conditionally expressed caJNK3 at 4 dpf in wildtype larvae. Larvae expressing caJNK3 in pLL neurons were immunolabeled with an anti-Lamp1 antibody and axon terminals were imaged. This analysis demonstrated that elevation of pJNK levels did not increase Lamp1 levels above controls (Figure 7A, 7B). Importantly, lysosome number and dynamics appeared normal in the presence of activated JNK, as Lysotracker red vital dye labeling was similar between caJNK3 expressing axons and non-expressing neighboring axons (Figure 7C, 7D).

Based on genetic work in *Drosophila*, JNK has been postulated to act as a “switch”, controlling anterograde vs. retrograde motor activity for cargo transport [38]. Thus, we asked whether Jip3-JNK interaction could be a potential regulator of directional lysosome transport. First, we used sequential imaging to determine if JNK3 and lysosomes were co-transported by co-expressing JNK3-mEos and Lamp1-mTangerine in pLL axons and imaging their transport at 2 dpf (N = 6; Video S9). This analysis demonstrated that only ∼19% of Lamp1-positive vesicles moving in the anterograde or retrograde direction were co-labeled with JNK3-mEos. Interestingly, 72% of JNK3 positive retrograde vesicles label with Lamp1-mTangerine, suggesting that, though lysosomes do not rely on JNK3 for their movement, JNK3 was transported with lysosomes towards the cell body.

Finally, we tested whether Jip3-JNK interaction had any function in lysosome transport, which, if disrupted, could lead to lysosome accumulation in axon terminals in the absence of Jip3. To address this, we assayed whether lysosome accumulation in *jip3^nl7^* mutants could be rescued by expressing Jip3ΔJNK (and Jip3 as a control) by RNA injection. For this assay, RNA was co-injected with the Lamp1-mTangerine DNA construct to visualize lysosomes in individual axons (see Figure 2E, 2F). Rescue score was determined as the average of the scores recorded by 2 blind, independent raters and was based on the ratio of punctate lysosomes (similar to wildtype in Figure 2E) vs. aggregates (as in mutants in Figure 2F). This analysis determined that Jip3ΔJNK was as effective as full-length Jip3 at suppressing lysosome accumulation in *jip3^nl7^* mutants (Figure 7E). We did not, however, observe complete rescue, potentially due to RNA degradation by 3 dpf. To complement this analysis, we implemented a DNA-based expression strategy that would allow expression of the rescue constructs at later stages. We expressed Jip3-mCherry and Jip3ΔJNK-mCherry in pLL axons using the *5kbneurod* promoter and assayed larvae for lysosome accumulation using Lamp1 immunolabeling at 4 dpf. Larvae were imaged live at 4 dpf to identify the axon terminals expressing these constructs and to identify mutant and wildtype siblings based on axonal phenotype of mCherry negative axons. Subsequently, larvae were individually immunolabeled for pJNK and Lamp1 and the same axon terminals were reimaged. Consistent with our previous results (see Figure 6), Jip3ΔJNK failed to rescue axon terminal swellings or pJNK accumulation in *jip3^nl7^* mutants but was capable of suppressing the elevation of Lamp1 levels similar to full-length Jip3 (Figure 7F–7I and data not shown; N = 5 out of 8 *jip3^nl7^* mutants injected with Jip3ΔJNK showed full rescue). Together, these data argue that Jip3-JNK interaction is not necessary for retrograde lysosome transport and supports a JNK-independent role for Jip3 in lysosome clearance from axon terminals.

### Jip3 functions in lysosome-dynein light intermediate chain (DLIC) association during retrograde lysosome transport

**Figure 8:**
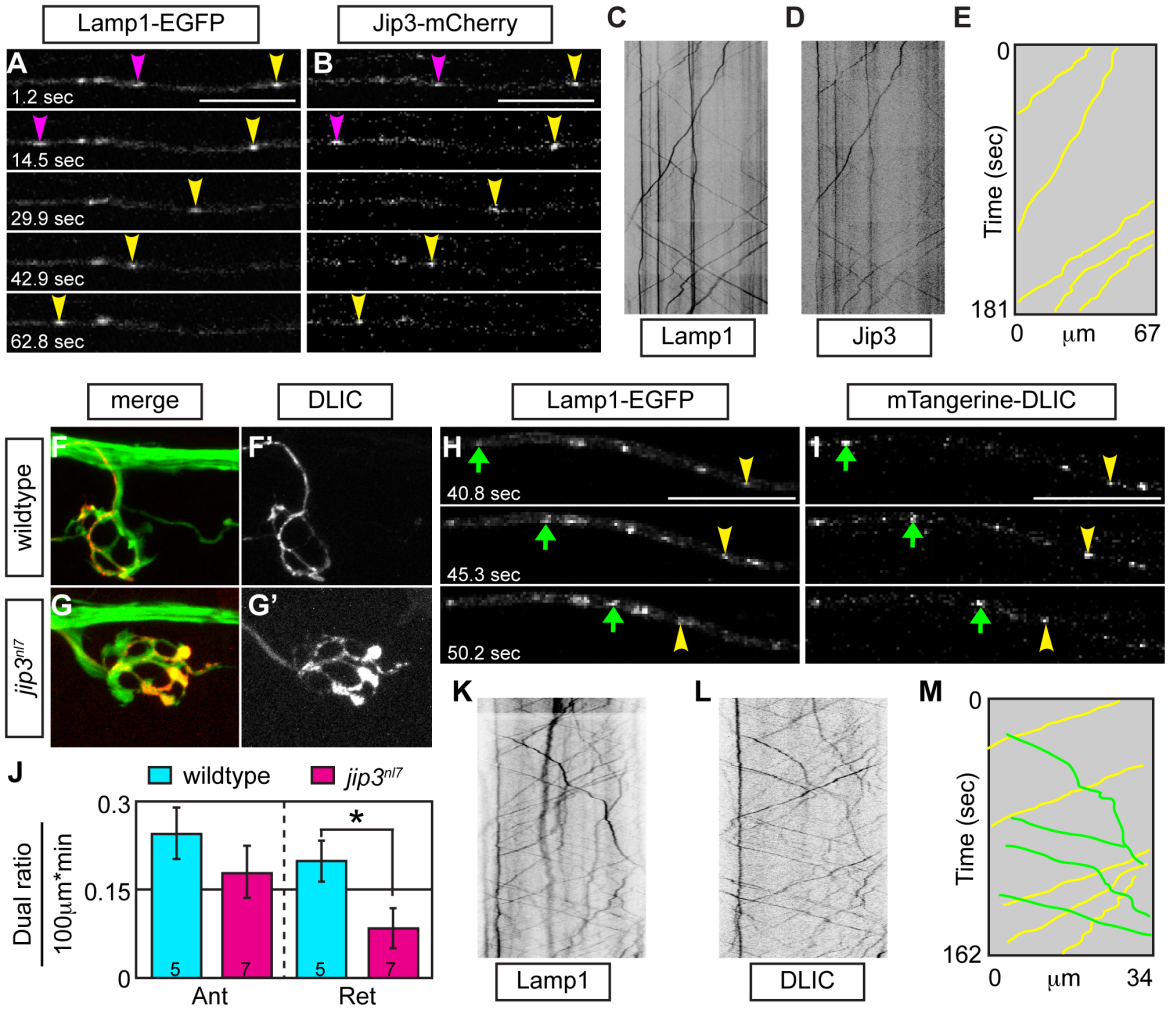
Jip3 scaffolds lysosomes to DLIC for retrograde transport. (A,B) Stills from a wildtype imaging session at 3 dpf in which Lamp1-EGFP (A) and Jip3-mCherry (B) co-transport was analyzed (Video S10). Pink and yellow arrowheads point to two retrograde Jip3/Lamp1 positive cargos. (C,D) Kymographs generated from this imaging session for individual cargos. (E) Schematized kymograph of co-transport. Yellow lines denote Jip3-positive lysosomes moving in the retrograde direction. (F,G) mTangerine-DLIC expression in a wildtype (F) and *jip3^nl7^* mutant (G) NM1 axon terminal at 3 dpf. (H,I) Stills from analysis of Lamp1 (H) and DLIC (I) co-transport at 3 dpf in a wildtype (Video S11). Green arrow-anterograde co-labeled puncta. Yellow arrowhead-DLIC positive lysosome undergoing retrograde transport. (J) The ratio of DLIC positive lysosomes moving in the retrograde direction was significantly decreased in *jip3^nl7^* mutants (ANOVA, *-*p*<0.05; Anterograde-Ant; Retrograde-Ret). (K–M) Kymographs from this imaging session and schematized kymograph depicting co-labeled anterograde lysosomes in green and retrograde in yellow.

In cultured cells, DLIC, a dynein accessory protein, functions in dynein-dependent lysosome transport [30]. As Jip3 has been shown to interact with DLIC [22], we hypothesized that Jip3 might serve as an adapter for lysosome-DLIC attachment during retrograde lysosome transport in axons. To ascertain whether Jip3 co-localized with moving lysosomes and could function in such a direct role, we performed sequential imaging of axons expressing both Jip3-mCherry and Lamp1-EGFP cargos at 2 and 3 dpf. Co-transport analysis revealed that Jip3 is present on lysosomes moving in the retrograde direction at both time-points (Figure 8A–8E; Video S10). Interestingly, the percentage of lysosomes that were transported in the retrograde direction labeled with Jip3 was higher at 3 dpf than at 2 dpf (2 dpf: 15%±3.8%, N = 5 vs. 3 dpf: 37%±4.2%, N = 4). This may indicate a differential reliance on Jip3 for the transport of this organelle beyond 2 dpf, leading to the decrease in lysosome retrograde transport frequency only after 2 dpf in *jip3^nl7^* (see Figure 2).

**Figure 9:**
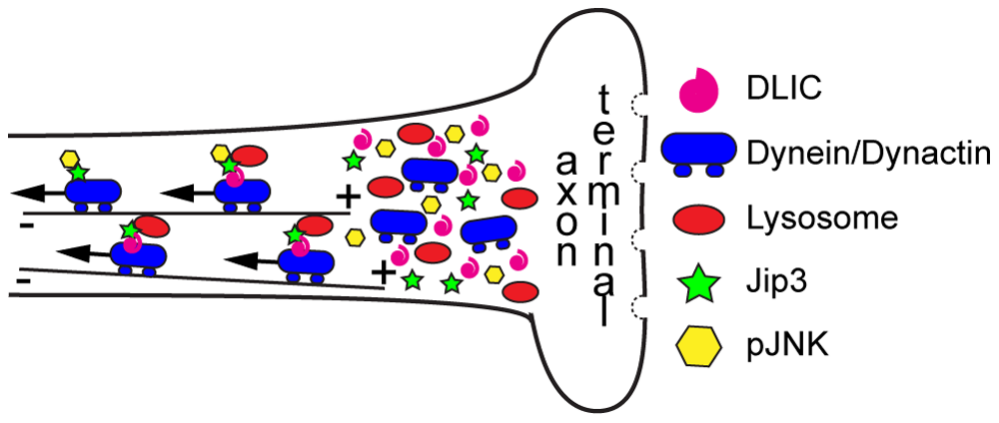
Model of Jip3's role in retrograde transport of lysosomes and pJNK. Our data support a model in which Jip3 (green star) serves as a necessary adapter for retrograde transport of pJNK (yellow hexagon) and lysosomes (red oval). This interaction serves to attach these cargoes to the dynein motor complex and, in the case of lysosomes, likely requires interaction with dynein light intermediate chain (DLIC). Global retrograde transport initiation is unaffected with loss of Jip3 as dynein heavy chain, dynactin and other dynein cargos (late endosomes and autophagosomes) do not accumulate in *jip3^nl7^* mutant axon terminals.

Finally, we co-expressed DLIC tagged with mTangerine (mTangerine-DLIC) and Lamp1-EGFP to characterize DLIC localization and co-transport with lysosomes and determine if this association is lost in *jip3^nl7^* mutants. At 3 dpf, mTangerine-DLIC localized to discrete puncta along the axon and in axon terminals in wildtype larvae (Figure 8F). In contrast, in *jip3^nl7^* mutants, DLIC accumulated in axon terminals, similar to lysosomes and pJNK (Figure 8G). Co-transport analysis of mTangerine-DLIC and Lamp1-EGFP cargos revealed a decrease in the ratio of DLIC-positive lysosomes moving in the retrograde direction in *jip3^nl7^* mutants (Figure 8H–8M; Video S11). This observation points to a failure of lysosome-dynein interaction during transport with loss of Jip3. Interestingly, there was a slight decrease in DLIC-Lamp1 vesicle co-transport in the anterograde direction as well in *jip3^nl7^* mutants suggesting that this complex may move bidirectionally. In summary, our data supports a model where the independent interaction of Jip3 with pJNK and lysosomes is required for the attachment of these cargoes to the dynein motor for clearance from axon terminals (Figure 9).

## Discussion

Our results revealed a novel role for Jip3 in retrograde axonal transport. We provided evidence that loss of Jip3 led to a decreased frequency of retrograde transport of an active kinase (pJNK) and lysosomes but not other components of the endosomal or autophagocytic system. We demonstrated that direct interaction of Jip3 and JNK was necessary to prevent pJNK accumulation and the axon terminal swellings characteristic of the *jip3^nl7^* mutant but had no effect on lysosome accumulation. Additionally, exogenous expression of activated JNK phenocopied the *jip3^nl7^* mutant axon terminal swellings but did not cause lysosome accumulation, providing evidence that high levels of active JNK cause this phenotype in a lysosome-independent manner. Finally, our co-transport analysis suggested that Jip3 directly facilitated lysosome interaction with the dynein motor through binding to the accessory protein DLIC. Given the decrease in frequency of cargo movement, the normal distribution of dynein components in *jip3^nl7^* mutant axon terminals, and the high rate of Jip3-lysosome and Jip3-JNK3 co-transport, we posit that Jip3 likely serves as an adapter protein that mediates attachment of these cargos to the dynein motor (Figure 9).

Jip3 has been implicated in anterograde axonal transport in several studies through its interaction with both Kinesin light chain and Kinesin heavy chain components of the Kinesin-1 motor [12], [13], [23]. We became interested specifically in Jip3's function in *retrograde* transport as *jip3^nl7^* demonstrated the unusual quality of extreme swellings in axon terminals, the end of the line for anterograde transport. A function for Jip3 in retrograde transport has indeed been posited by Cavalli et al. as they demonstrated that Jip3 co-localized with pJNK distal to nerve ligation and co-purified from similar membrane fractions as dynein components [15]; however, our study is the first to provide conclusive evidence that Jip3 is required for retrograde transport of pJNK, as pJNK accumulates in axon terminals in *jip3^nl7^* mutants, Jip3 and JNK3 are co-transported, and direct Jip3-JNK interaction is functionally required for pJNK retrograde transport. Thus, our work identifies pJNK as a Jip3-dependent retrograde cargo. In addition, through the implementation of our *in vivo* imaging approach, we found that the frequency of retrograde JNK3 transport was decreased with loss of Jip3, but the processivity of the motor (reflected by run length) and velocity of movement were unchanged. This data, in combination with previous biochemical studies of Jip3-JNK and Jip3-dynein interaction [15], provide strong evidence that Jip3 functions as an adapter for pJNK, linking it to the dynein complex for transport, while not affecting motor movement itself.

Using a combination of immunolabeling and *in vivo* imaging techniques, we further show that Jip3 is necessary for retrograde transport of lysosomes through interaction with the dynein accessory protein DLIC. DLIC has been shown to be an important mediator of dynein-based lysosome movement in culture systems [30] and was shown to biochemically interact with Jip3 in another system [22]. Thus, Jip3 could provide a link between lysosomes and dynein through its interaction with DLIC. In support of this, Jip3 is co-transported with lysosomes, the retrograde transport velocities for Jip3 alone were highly similar to those observed for lysosomes, and DLIC-lysosome co-transport was significantly decreased in *jip3^nl7^* mutants. Together, these data provides strong evidence that Jip3 serves as an important adapter protein for lysosome-DLIC interaction and subsequent retrograde lysosome transport. Notably, Jip3 was implicated in the anterograde transport of DLIC to axon terminals in *C. elegans* [22]. However, instead of a decrease, we observed increased levels of DLIC in *jip3^nl7^* axon terminals, arguing that this Jip3 function may not be conserved in vertebrates or is compensated for by another member of the Jip family [39].

Elevated levels of activated JNK, lysosome accumulation and axonal dysmorphology have been co-associated with neurodegenerative disorders [40]. Interestingly, though our studies indicated that Jip3-JNK interaction was not required for lysosome retrograde transport, JNK3 was frequently present on lysosomes moving in the retrograde direction, suggesting that Jip3 could serve to attach both cargos to the dynein motor simultaneously. Furthermore, our results point to a lysosome-independent etiology of axon terminal swellings in *jip3^nl7^* mutants. Evidence to support a lysosome-independent mechanism includes: 1) the ability to induce axonal swellings without lysosome accumulation by exogenous expression of constitutively active JNK; 2) the absence of axon morphological changes following expression of an inactivated form of the constitutively active JNK; and 3) rescue of lysosome accumulation, but not pJNK levels or axonal swellings, in *jip3^nl7^* mutant axon terminals by Jip3ΔJNK expression. Thus, our work provides evidence that axonal swellings can occur downstream of this active kinase without causing concomitant accumulation of organelles in the autolysosomal pathway. The exact etiology of axonal swellings in *jip3^nl7^* mutants due to elevated levels of activated JNK remains to be determined.

Importantly, *jip3^nl7^* mutants did not exhibit a global disruption of retrograde axonal transport, which would indirectly lead to cargo accumulations. Evidence supporting the specificity of transport disruptions includes: 1) absence of the accumulation of other cargo (late endosomes, autophagosomes, and signaling endosomes) in *jip3^nl7^* axon terminals; and 2) normal localization of dynein heavy chain and p150^glued^ in *jip3^nl7^* axon terminals, indicating that dynactin-based initiation of dynein transport is not hindered [9], [10]. Thus, our data supports a direct role for Jip3 as an adapter for the transport of two specific retrograde cargos, pJNK and lysosomes.

In summary, our data demonstrate novel and separate roles for Jip3 in the retrograde axonal transport of activated JNK and lysosomes. It is tempting to speculate that Jip3-dependent retrograde clearance of activated JNK may be a novel and crucial strategy for the removal of this active kinase from axon terminals, bypassing traditional phosphatase pathways. Furthermore, we show that enhanced JNK activity can indeed cause axon terminal swellings, similar to those observed in the *jip3^nl7^* mutant, in the absence of lysosome accumulation. Thus, we have shown that there can be an independent etiology for these tightly coupled events observed in disease models. The similarities between the axonal swellings, high levels of pJNK, and accumulation of lysosomes in *jip3^nl7^* and neurodegenerative diseases such as Alzheimer's Disease points to an intricate relationship between these phenotypes during pathogenesis. Our studies begin to unravel how Jip3-dependent regulation of retrograde axonal transport may underlie or modulate such disease states.

## Materials and Methods

### Zebrafish husbandry

Adult *AB and WIK zebrafish and *AB/WIK hybrids were maintained at 28.5°C and staged as described [41]. Embryos were derived from natural matings or *in vitro* fertilization, raised in embryo media, and developmentally staged using previously established methods [42]. Strains utilized included TgBAC(*neurod:EGFP*)*^nl1^* [16], TgBAC(*phox2b:EGFP*)*^w37^* [18], TgBAC(*neurog:DsRed*)*^nl6^*, TgBAC(*foxd3:EGFP*)*^nl5^* transgenics and *mitfa^w2^* [43], and *mapk8ip3^nl7^* (*jip3^nl7^*) mutants.

### Generation of transgenic lines

We used Escherichia coli-based homologous recombination to modify a *neurog1*- and *foxd3*-containing bacterial artificial chromosome (BAC) clones [44]. The *neurog1* BAC clone zK171N3 contains 63.8 kb of upstream and 106.1 kb of downstream sequence of *neurog1*, while the *foxd3* BAC clone zC137J12 contains 66.2 kb of upstream and 122.1 kb of downstream sequence of *foxd3* (http://www.sanger.ac.uk/Projects/D_rerio/mapping.shtml). After recombination, the modified BAC clones contained DSRedExpress-1 and EGFP positioned at the endogenous start site of *neurog1* or *foxd3*, respectively. The accuracy of recombination was evaluated by PCR, sequencing, and analysis of transient expression. To obtain germline transgenics, we microinjected 20–80 pg of BAC DNA (linearized with Srf I for *neurog1* BAC and supercoiled for *foxd3* BAC) into zebrafish zygotes, raised injected fish to adulthood, and screened their progeny for reporter gene expression. The germline transmission rate was 2.3% for *neurog1* BAC and 1.4% for the *foxd3* BAC. The TgBAC(*neurog1:DSRed*)*^nl6^* and TgBAC(*foxd3:EGFP*)*^nl5^* strains have been outcrossed for multiple generations and transfmitted the transgenes in a Mendelian manner.

### ENU mutagenesis and positional cloning

The *jip3^nl7^* mutant was identified in a standard three-generation N-ethyl-N-nitrosourea (ENU) mutagenesis screen [45], [46]. For this screen, TgBAC(*neurod:EGFP*)*^nl1^*-positive larvae were screened at 4 dpf for axon truncation and the presence of axonal swellings under epifluorescence. For genetic mapping, heterozygous carriers of *jip3^nl7^* on a polymorphic *AB/WIK background were incrossed to produce homozygous, heterozygous and wildtype progeny. Initial chromosome assignment was done by bulk segregate analysis of DNA pools from 20 wildtype and 20 mutant individuals using microsatellite markers (http://zfin.org/ZFIN). Flanking regions were identified using individual wildtype and mutant larva and markers z15457, z21697, and a designed marker, CA50 (forward: 5′-TTACACACTTTCAGCCTGTC, reverse: 5′-CCTTTATGCCACGGTCACA).

### Genomic DNA isolation, cDNA generation, gene cloning, and genotyping

Genomic DNA was isolated from larvae by incubating it overnight at 55°C in PCR Extraction Buffer (10 mM Tris pH = 8, 2 mM EDTA, 0.2% Triton X-100, 200 µg/ml Proteinase K). Total RNA was isolated from larvae using Trizol according to the manufacture's protocol (Invitrogen) and cDNA was generated using Superscript II reverse transcriptase and oligo dT primers (Invitrogen). The full *mapk8ip3* (*jip3*) cDNA was amplified using following primers (forward: 5′-CGTTAAACGAGCTTCGGACA, reverse: 5′-GCGTGTCACTTTGAGTTTGG) based on the predicted sequence and subsequently entered into GenBank (KC170712). Full-length *jnk3* was amplified using primers (forward: 5′-ATGAACAGACGTTTCTTATATAACTGC, reverse: 5′-CACGGCTGCACCTGCGCTG) designed against the annotated sequence (NM 001037701). Full-length dynein light intermediate chain was amplified using primers (forward: 5′-TGTCACTCAAGCCTGCGAAG, reverse: 5′-GGATTTGTCGTTTTCAGCAG) designed against the annotated sequence (NM 001017669). To genotype *jip3^nl7^* carriers, a 385 bp region around the mutation was amplified from genomic DNA by PCR using annealing T = 55°C and the following primers (forward: 5′-TTTGTCTGTTGAAATTGCT, reverse: 5′-ACGGTCCATACCCATGATT). PCR products were then digested with SpeI, as the single nucleotide change generates this restriction site in the *jip3^nl7^* allele, producing two bands, 243 and 142 bp.

### *In situ* hybridization, immunohistochemistry, TUNEL, and Lysotracker staining

RNA *in situ* hybridization was performed as described [47]. Digoxygenin-labeled antisense RNA probes were generated for *jip3* and *jnk3* using the full-length cDNA cloned. Whole mount immunohistochemistry was performed following established protocols [48]. The following antibodies were used: anti-GFP (1∶1000; Invitrogen #A11122), anti-pJNK (1∶100; Cell Signaling #9251S), anti-tJNK (1∶100; Cell Signaling #9252), anti-p150^glued^ (1∶100; Signal Transduction Labs #610473), anti-dynein heavy chain (1∶100; gift of R. Vallee; [49]), anti-Rab7 (1∶100; Sigma #R8779), anti-Lamp1 (1∶100; Developmental Studies Hybridoma Bank), anti-LC3 (1∶100; Novus #NB100-2331), anti-TrkB (1∶100; Santa Cruz Biotechnology #sc-12) and Alexa-488/568/647 (1∶750; Invitrogen). Antibodies not used previously in zebrafish were validated by Western blot analysis (see below: Figure S3I–S3L; Rab7–24 kD, LC3–14.5 kD, TrkB-69 kD and 18 kD, Lamp1–27 kD). For TUNEL labeling, embryos were processed as previously described [50] with minor modifications according to the manufacturer's instructions (In situ cell death kit, Roche). For Lysotracker red vital dye staining, 4–5 dpf larvae were incubated in Lysotracker red (1∶10,000; Invitrogen) for 15 minutes in embryo media, washed briefly, embedded in 1.2% low-melt agarose, and imaged. All fluorescently labeled embryos were imaged using a FV1000 laser scanning confocal system (Olympus). Brightfield or Nomarski microscopy images were collected using a Zeiss Imager Z1 system. Images were processed using ImageJ software [51]. Brightness and contrast were adjusted in Adobe Photoshop and figures were compiled in Adobe Illustrator.

### Western blotting

For western blot analysis, protein was isolated from wildtype and *jip3^nl7^* 3 dpf larvae by homogenizing individuals in extraction buffer (150 mM NaCl, 50 mM Tris pH = 7.4, 5 mM EDTA, 0.05% NP40, 25 mM NaF, 10 mM Na_3_VO_4_, 1 mM DTT, 10 µL/mL protease inhibitor) at a ratio of 4 µL buffer per embryo. The equivalent of 4 embryos was run in each lane on a 12% SDS-PAGE gel and transferred onto a PVDF membrane (Millipore). Primary antibodies were applied overnight: anti-pJNK (1∶1000; Cell Signaling #9251S), anti-tJNK (1∶1000; Cell Signaling #9252), anti-p150^glued^ (1∶1000; Signal Transduction Labs #610473), anti-dynein heavy chain (1∶1000; gift of R. Vallee; [49]), anti-Rab7 (1∶2000; Sigma #R8779), anti-Lamp1 (1∶4000; Developmental Studies Hybridoma Bank), anti-LC3 (1∶500; Novus #NB100-2331), anti-TrkB (1∶100; Santa Cruz Biotechnology #sc-12), and anti-p-cJun (1∶1000; Cell Signaling 9164S). After washing, an anti-rabbit*HPR, anti-mouse*HRP, or anti-rat*HRP secondary (1∶10,000; Jackson Immuno) was applied for 90 minutes. Protein was visualized using SuperSignal West Pico Chemiluminescent Substrate according to the manufacture's specification (Thermo Scientific). If necessary, the blot was then stripped with 25 mM glycine (pH = 2.5) and re-probed with rabbit anti-α-actin (1∶10,000; Sigma).

### Generation of caJNK3 and caJNK3-IA constructs and heat-shock activation

To generate constitutively active JNK3 that could be activated in a temporally specific manner, we fused MKK7 to JNK3 and placed this fusion behind a heat-shock inducible promoter (*hsp70:mkk7-JNK3-egfp*, referred to as *caJNK3* in the text). To generate an inactive form of the same construct (referred to as *caJNK3-IA* in the text), two amino acids were mutated (T221A and Y223F) to render JNK3 not able to be phosphorylated, which is required for its activity [37]. For induction of transcription of both constructs, 4 dpf larvae injected with 10 pg of the *caJNK3* or *caJNK3-IA* constructs were heat-shocked at 38°C for 1 hour. Larvae were then transferred to 28.5°C prior to analysis.

### Axon transport analysis

Zygotes were injected with plasmid DNA encoding fluorescently tagged cargos of interest with expression driven by the *5kbneurod* promoter [26]. At 30 hpf, 2 dpf, or 5 dpf, embryos or larvae were sorted under epifluorescence to identify individuals with tagged cargo expression in a few cells of the pLL ganglion. For imaging, embryos were mounted in 1.2% low melting point agarose on a glass coverslip, submerged in embryo media containing 0.02% tricaine and imaged using a 60X/NA = 1.2 water objective on an upright Fluoview1000 confocal microscope (Olympus). For each embryo, a region of interest (30–200 µm) was selected in the pLL nerve in which a long stretch of axon was observable in a single plane. Scans were taken at the fastest possible speed (3–5 frames per second) for 600 to 2500 frames. Embryos were subsequently released from agarose and processed for genotyping. For co-transport, embryos expressing both constructs in a single cell were selected and imaged as described above using sequential imaging of the 488 and 568 nm excitation channels. 600 frames were collected at 2–3 frames per second.

Transport parameters were analyzed using kymograph analysis in the MetaMorph software package (Molecular Devices, Inc.). Kymographs were generated from each imaging session and used to determine distance moved in individual bouts of movement (uninterrupted straight lines) and velocity of movement (slope of uninterrupted straight lines). Typically, 10–50 traces were analyzed in each kymograph and these were averaged within individual embryos for statistical analysis. The number of particles moving in each direction was estimated based on traces on the kymographs and then normalized to length of axonal segment and total imaging time.

### Axotomy and image acquisition

Five day old zebrafish larva (*neurod:EGFP* carriers) were anesthetized in 0.02% tricaine (MS-222; Sigma) and embedded in 3% methylcellulose on a slide. Pulled thick-walled glass capillaries were used to sever the nerve between NMs 2 and 3. Slides were immersed in Ringer's solution (116 mM NaCl, 2.9 mM KCl, 1.8 mM CaCl_2_, 5 mM HEPES pH = 7.2, 1% Pen/Strep) and incubated at 28.5°C for 3 hours. Larva were then collected and immunolabeled for pJNK or tJNK and EGFP. Details of image and statistical analyses are described below.

### Quantification of immunofluorescence

For analysis of pJNK and tJNK intensity in axon terminals and after nerve injury, individuals were immunolabeled as described above. For consistency of labeling, larvae that were directly compared were processed in the same batch. Confocal Z-stacks (0.5 µm between planes) were taken of the area of interest using a 40X/NA = 1.3 oil objective with identical settings. Images were analyzed using ImageJ [51]. For fluorescent intensity measurements of pJNK or tJNK in wildtype and mutant axon terminals, summed projections of the regions of interest were generated only through regions that contained the *neurod:EGFP* signal and converted to 8 bit in ImageJ. In the pLL nerve injury analysis, a 30 µm, *neurod:EGFP*-positive region encompassing the proximal or distal edge of the severed axon was selected and summed projections through only this segment were compiled for analysis. By restricting our analysis to the *neurod:EGFP* axons we eliminated a majority of the fluorescent signal from the surrounding tissue. Prior to statistical comparison, the mean background fluorescent intensity, measured in a region adjacent to the NM axon terminal or injury site, was subtracted from the values generated.

For analysis of pJNK levels in the DNA rescue experiment, axon terminals expressing Jip3-mCherry or Jip3ΔJNK-mCherry (typically innervating half a NM) and control terminals not expressing these constructs (the alternate half of the NM) were outlined in similar summed confocal projections and the mean fluorescent intensity was measured. The ratio of pJNK fluorescence in the axons expressing the rescue construct to those not expressing the rescue construct were compared for statistical analysis.

### Statistical analysis

Statistical analysis was performed using the JMP software package. Data suitable for parametric analysis were analyzed using ANOVA, with Tukey-Kramer highly significant difference post-hoc contrasts for more than two variables. Data not suitable for parametric analysis were analyzed using Wilcoxon rank-sum analysis. Ordinal data was analyzed using Chi Square test. In all cases, data from individual embryos was averaged prior to analysis making each N equivalent to an embryo.

### Ethics statement

All animal work was approved by and conducted according to guidelines of the Oregon Health & Science University IACUC.

## Supporting Information

Figure S1 pLL nerve abnormalities in *jip3^nl7^* were not due to cell death, general cytoskeletal defects or glial deficits. (A–D) Cell death in the pLL ganglion was examined by TUNEL assay at 30 hpf and 2 dpf. Confocal projections of the pLL ganglion showed that TUNEL labeling (red in merge, white in single channel) was not elevated in *jip3^nl7^* during (30 hpf) or after (2 dpf) nerve extension. Expression of the *neurod:EGFP* transgene marks the pLL ganglion. (E) Quantification of TUNEL-positive cells in the pLL ganglia of wildtype and mutant embryos. (F–I) Immunolabeling with an antibody against a neurofilament associated antigen demonstrated no deficit in the pLL nerve of *jip3^nl7^* at 2 and 5 dpf. (J–M) Similarly, analysis of microtubule density in the pLL nerve with an antibody against α-tubulin revealed no significant changes in the *jip3^nl7^*. Arrow indicates hair cell stereocilia which contain high levels of tubulin. The distal nerve at NM4 is shown for F–M. (N–Q) Glial cell occupation of the pLL nerve was analyzed using a combination of two transgenic lines, TgBAC(*foxd3:EGFP*)*^nl5^* (glia) and TgBAC(*neurog1:DsRed*)*^nl6^* (nerve). Just after nerve extension (2 dpf), glial occupation of the pLL nerve was normal in *jip3^nl7^*. The distal portion of the pLL nerve at NM4 is shown in P and Q. (R,S) Myelination of the pLL nerve was also normal at 5 dpf in *jip3^nl7^* as assayed by antibodies against MBP (myelin basic protein). The pLL ganglion is outlined. Scale bars in A–D, F–M and R,S are 25 µm; N–Q are 100 µm.
(TIF)

Figure S2 Membrane-bound cargo accumulated in *jip3^nl7^* axon terminals due to failed retrograde transport. (A,B) ssNPY-mCherry (red) accumulated in mutant (arrowheads), but not wildtype axon terminals at 5 dpf. Asterisk indicates portion of the nerve occluded by a pigment cell. The pLL axons were visualized by expression of the *neurod:EGFP* transgene. (C–F) Representative still images from Videos S2 and S3 and kymographs from transport analysis in 5 dpf larvae. (G) Analysis of anterograde and retrograde ssNPY-mCherry particle movements at 2 dpf revealed a decrease in anterograde and retrograde cargo movement (ANOVA; **-*p*<0.005). (H, I) Other transport parameters, including distance moved in individual movement bouts and movement velocity, were unchanged in *jip3^nl7^*. Scale bars = 10 µm. Number of embryos analyzed is indicated on each bar of the graph.
(TIF)

Figure S3 Lysosomes, but not late endosomes, signaling endosomes or autophagosomes, accumulated in *jip3^nl7^* axon terminals. (A–H) The density of late endosomes, autophagosomes, TrkB, and lysosomes were assayed in wildtype and *jip3^nl7^* axon terminals by immunolabeling (A–F) and live imaging (G,H). (A,B) Rab7, a marker of late endosomes, was unchanged in *jip3^nl7^* axon terminals. (C,D) LC3, a marker of autophagosomes, was unchanged in *jip3^nl7^* axon terminals (yellow arrow). Note the high LC3 expression in NM support cells (dotted outline). (E,F) TrkB levels were slightly decreased in *jip3^nl7^* axon terminals. (G,H) Lysotracker red staining at 5 dpf revealed elevated levels of lysosomes in *jip3^nl7^* axon terminals (red arrowhead). Asterisk points out representative hair cells. This cell type has high Lysotracker labeling due to the large number of acidic vesicles. (I–L) Western blot analyses of 3 dpf embryo lysates demonstrate the specificity of the antibodies used. Arrows indicate bands corresponding to those that match the size of the predicted zebrafish protein orthologs.
(TIF)

Figure S4 Total JNK levels were not elevated in *jip3^nl7^*. (A–D) Total JNK (tJNK) levels were unchanged in *jip3^nl7^*axon terminals at 2 and 5 dpf. (E,F) Western blot analysis of 3 dpf whole embryo extracts indicated overall levels of pJNK (E) and tJNK (F) were not changed in *jip3^nl7^* (*j*) compared to wildtype (w). α-actin control below. Scale bars = 10 µm.
(TIF)

Figure S5 Components of the dynein retrograde motor complex were present at *jip3^nl7^* axon terminals. (A–E) Immunolabeling for two components of the dynein motor complex, p150^glued^ (A–D; 2 and 5 dpf) and dynein heavy chain (E,F; 4 dpf) demonstrated that levels and distribution of these dynein motor components in axon terminals were similar between *jip3^nl7^* and wildtype controls. *neurod:EGFP* transgene carriers were used to label the pLL nerve (green). The middle portion of the pLL nerve at NM3 is shown in A–F. (G) Western blot analysis of 4 dpf whole embryo extracts demonstrated the specificity of the respective antibodies (arrow indicates DHC band based on predicted molecular weight).
(TIF)

Figure S6 *jnk3* is expressed in the peripheral and central nervous systems of the zebrafish embryo. (A) *In situ* hybridization for *jnk3* at 30 hpf revealed expression in the central and peripheral nervous system, including the pLL ganglion (inset). (B) *jnk3* expression in these regions, including the pLL ganglion (inset), persisted at 2 dpf.
(TIF)

Figure S7 Jip3-JNK interaction did not regulate axon extension. Injection of mRNA encoding both full-length Jip3 and Jip3 lacking the JNK binding domain (Jip3ΔJNK) rescued axon length in *jip3^nl7^*. Fraction of embryos expected to have truncated nerves in the respective crosses is indicated by the dashed line (50% in the homozygous X heterozygous cross and 25% in the heterozygous incross). Number of embryos analyzed and amount of DNA injected is indicated for each bar.
(TIF)

Video S1 Jip3-mCherry transport in the pLL nerve. Jip3-mCherry is actively transported in the anterograde (left to right) and retrograde (right to left) direction in a proximal segment of a pLL axon. The pink arrow denotes a particle moving in the anterograde direction while the yellow arrow points to a retrograde particle. The 2 dpf embryo is oriented with head to the left, dorsal up. Scale bar = 10 µm. Time is in seconds.
(MP4)

Video S2 ssNPY-mCherry was actively transported in the pLL nerve of a wildtype larva. Movie of a 5 dpf larva with head to the left, dorsal up, showing high levels of anterograde and retrograde transport in 3 axons of the pLL nerve. Scale bar = 10 µm. Time is in seconds.
(MP4)

Video S3 ssNPY-mCherry transport was hindered in *jip3^nl7^*. Some anterograde (pink arrow) but no retrograde ssNPY-mCherry transport is apparent in this movie of a single pLL axon in a *jip3^nl7^* mutant at 5 dpf. Scale bar is 10 µm. Time is in seconds.
(MP4)

Video S4 Lamp1-mTangerine labeled lysosomes were transported in the pLL nerve. Wildtype zebrafish expressing Lamp1-mTangerine in 2 axons of the pLL nerve at 3 dpf. Anterograde transport is from left to right. Scale bar = 10 µm. Note the high level of transport in both directions. Time is in seconds.
(MP4)

Video S5 Retrograde lysosome transport frequency was decreased in *jip^nl7^*. Movie of a 3 dpf *jip3^nl7^* mutant embryo expressing Lamp1-mTangerine in 2 axons of the pLL nerve. Note the high level of anterograde transport (left to right) as opposed to low retrograde transport (right to left). Scale bar = 10 µm.
(MP4)

Video S6 Anterograde and retrograde transport of JNK3-mEos in a wildtype pLL axon. At 2 dpf, JNK3 is transported in both directions in a proximal portion of a pLL axon. Yellow arrow indicates a JNK3-mEos positive particle moving in the retrograde direction and the pink arrow denotes an anterogradely moving particle. Scale bar = 10 µm. Time is in seconds.
(MP4)

Video S7 Retrograde JNK3-mEos transport frequency was decreased in *jip3^nl7^*. JNK3-mEos transport analysis in the proximal portion of a pLL axon at 2 dpf in *jip3^nl7^* demonstrates the dramatic reduction in the number of JNK3-mEos positive, retrograde cargo (arrowhead). Scale bar = 10 µm. Time is in seconds.
(MP4)

Video S8 JNK3 was highly co-transported with Jip3 in the retrograde direction. JNK3-mEos and Jip3-mCherry co-transport was analyzed by sequential live imaging in the proximal portion of a pLL axon at 2 dpf. Yellow arrowhead points to a JNK3-Jip3 positive vesicle moving in the retrograde direction. Merge (top), Jip3-mCherry (middle), JNK3-mEos (bottom) panels are shown. Scale bar = 10 µm. Time is in seconds.
(MOV)

Video S9 Lysosomes were transported largely in the absence of JNK3 in the anterograde and retrograde direction. Sequential imaging of a proximal segment of a pLL axon at 2 dpf in which Lamp1-mTangerine and JNK3-mEos were expressed. Though only a small subset of lysosomes (marked by Lamp1) were also positive for JNK3 in the retrograde pool, a large percentage of JNK3 that was moving in the retrograde direction traveled with lysosomes. Arrowhead indicates a JNK3 positive lysosome moving in the retrograde direction. Merge (top), Lamp1-mTangerine (middle), JNK3-mEos (bottom) panels are shown. Scale bar = 10 µm. Time is in seconds.
(MOV)

Video S10 Lysosomes were co-transported with Jip3 in pLL axons. Sequential imaging of Jip3-mCherry and Lamp1-EGFP in a pLL axon of a wildtype 3 dpf zebrafish larva. Pink and yellow arrowheads denote two individual lysosomes moving in the retrograde direction that are positive for Jip3. The green arrowhead indicates a Lamp1-positive lysosome moving in the anterograde direction that is not labeled by Jip3. Merge (top), Lamp1-EGFP only (middle) and Jip3-mCherry (bottom) panels are shown. Scale bar is 10 µm. Time is in seconds.
(MOV)

Video S11 Lysosome-DLIC co-transport in a wildtype pLL axon. Sequential imaging of Lamp1-EGFP and mTangerine-DLIC in a pLL axon of a wildtype 3 dpf larva demonstrates considerable but not complete co-transport of lysosomes with this dynein accessory protein. The green arrow points to a lysosome moving in the anterograde direction that is also positive for DLIC. The yellow arrowhead points to a Lamp1-DLIC positive lysosome moving in the retrograde direction. Merge (top), Lamp1-EGFP (middle) and mTangerine-DLIC (bottom) panels are shown. Scale bar = 10 µm. Time is in seconds.
(MOV)
