# Supplementary figures and images for: JNK-Interacting Protein 3 Mediates the Retrograde Transport of Activated c-Jun N-Terminal Kinase and Lysosomes
Source: PLoS Genet. 2013 Feb 28;9(2):e1003303. doi: 10.1371/journal.pgen.1003303 (PMC3585007; doi:10.1371/journal.pgen.1003303)

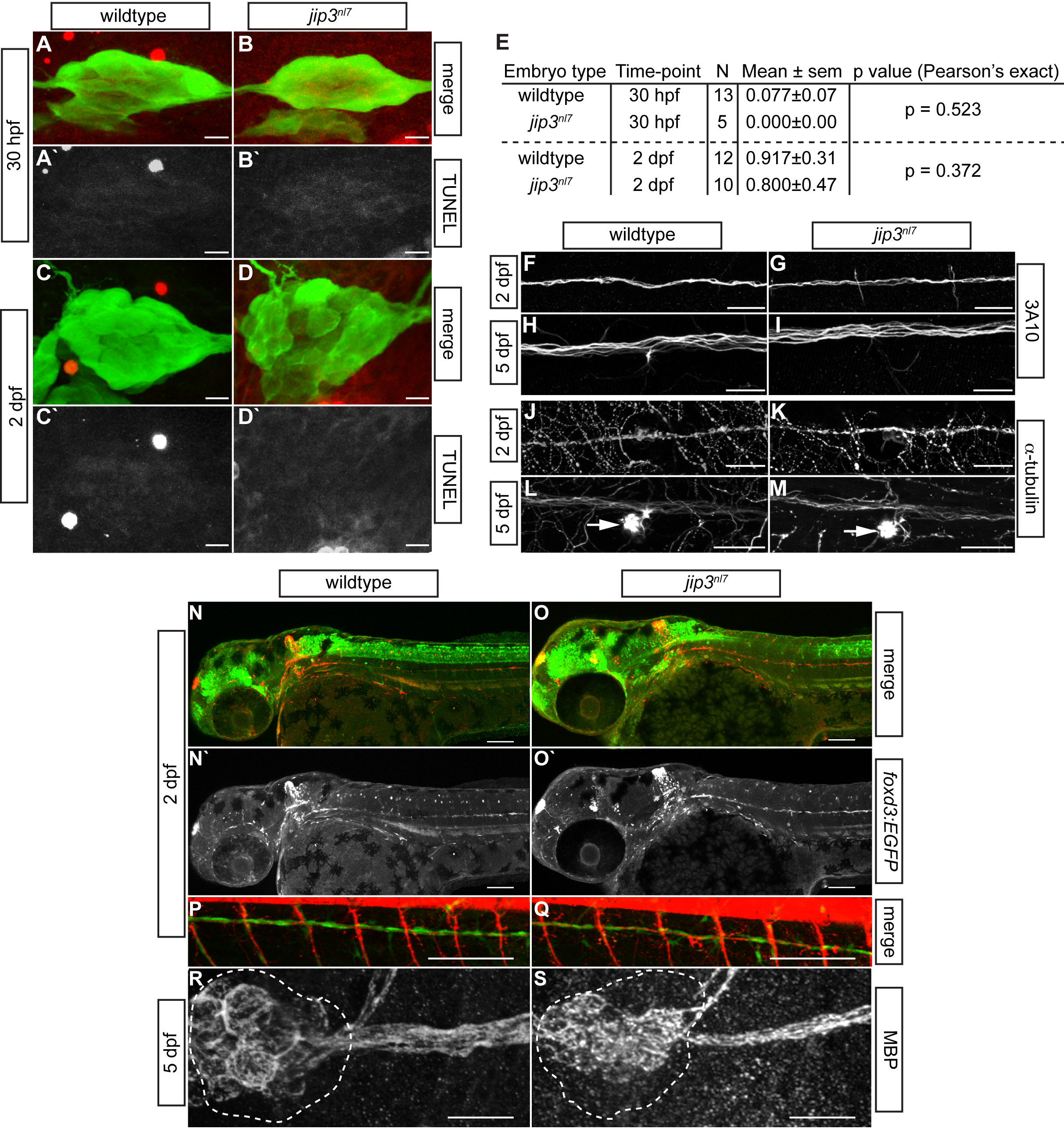

Supplement: Figure S1 — pLL nerve abnormalities in jip3nl7 were not due to cell death, general cytoskeletal defects or glial deficits. (A–D) Cell death in the pLL ganglion was examined by TUNEL assay at 30 hpf and 2 dpf. Confocal projections of the pLL ganglion showed that TUNEL labeling (red in merge, white in single channel) was not elevated in jip3nl7 during (30 hpf) or after (2 dpf) nerve extension. Expression of the neurod:EGFP transgene marks the pLL ganglion. (E) Quantification of TUNEL-positive cells in the pLL ganglia of wildtype and mutant embryos. (F–I) Immunolabeling with an antibody against a neurofilament associated antigen demonstrated no deficit in the pLL nerve of jip3nl7 at 2 and 5 dpf. (J–M) Similarly, analysis of microtubule density in the pLL nerve with an antibody against α-tubulin revealed no significant changes in the jip3nl7. Arrow indicates hair cell stereocilia which contain high levels of tubulin. The distal nerve at NM4 is shown for F–M. (N–Q) Glial cell occupation of the pLL nerve was analyzed using a combination of two transgenic lines, TgBAC(foxd3:EGFP)nl5 (glia) and TgBAC(neurog1:DsRed)nl6 (nerve). Just after nerve extension (2 dpf), glial occupation of the pLL nerve was normal in jip3nl7. The distal portion of the pLL nerve at NM4 is shown in P and Q. (R,S) Myelination of the pLL nerve was also normal at 5 dpf in jip3nl7 as assayed by antibodies against MBP (myelin basic protein). The pLL ganglion is outlined. Scale bars in A–D, F–M and R,S are 25 µm; N–Q are 100 µm. (TIF) [file pgen.1003303.s001.tif]

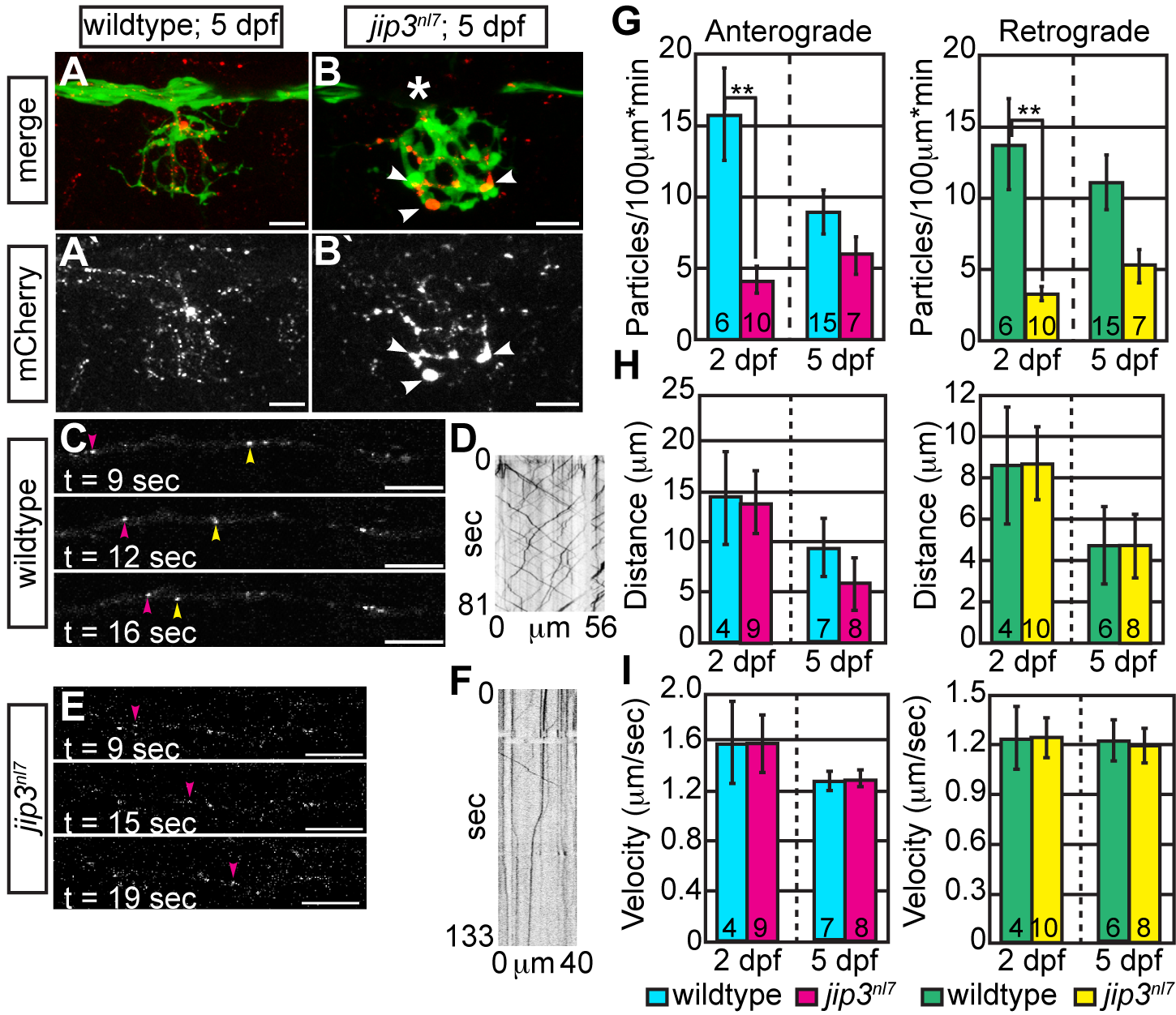

Supplement: Figure S2 — Membrane-bound cargo accumulated in jip3nl7 axon terminals due to failed retrograde transport. (A,B) ssNPY-mCherry (red) accumulated in mutant (arrowheads), but not wildtype axon terminals at 5 dpf. Asterisk indicates portion of the nerve occluded by a pigment cell. The pLL axons were visualized by expression of the neurod:EGFP transgene. (C–F) Representative still images from Videos S2 and S3 and kymographs from transport analysis in 5 dpf larvae. (G) Analysis of anterograde and retrograde ssNPY-mCherry particle movements at 2 dpf revealed a decrease in anterograde and retrograde cargo movement (ANOVA; **-p<0.005). (H, I) Other transport parameters, including distance moved in individual movement bouts and movement velocity, were unchanged in jip3nl7. Scale bars = 10 µm. Number of embryos analyzed is indicated on each bar of the graph. (TIF) [file pgen.1003303.s002.tif]

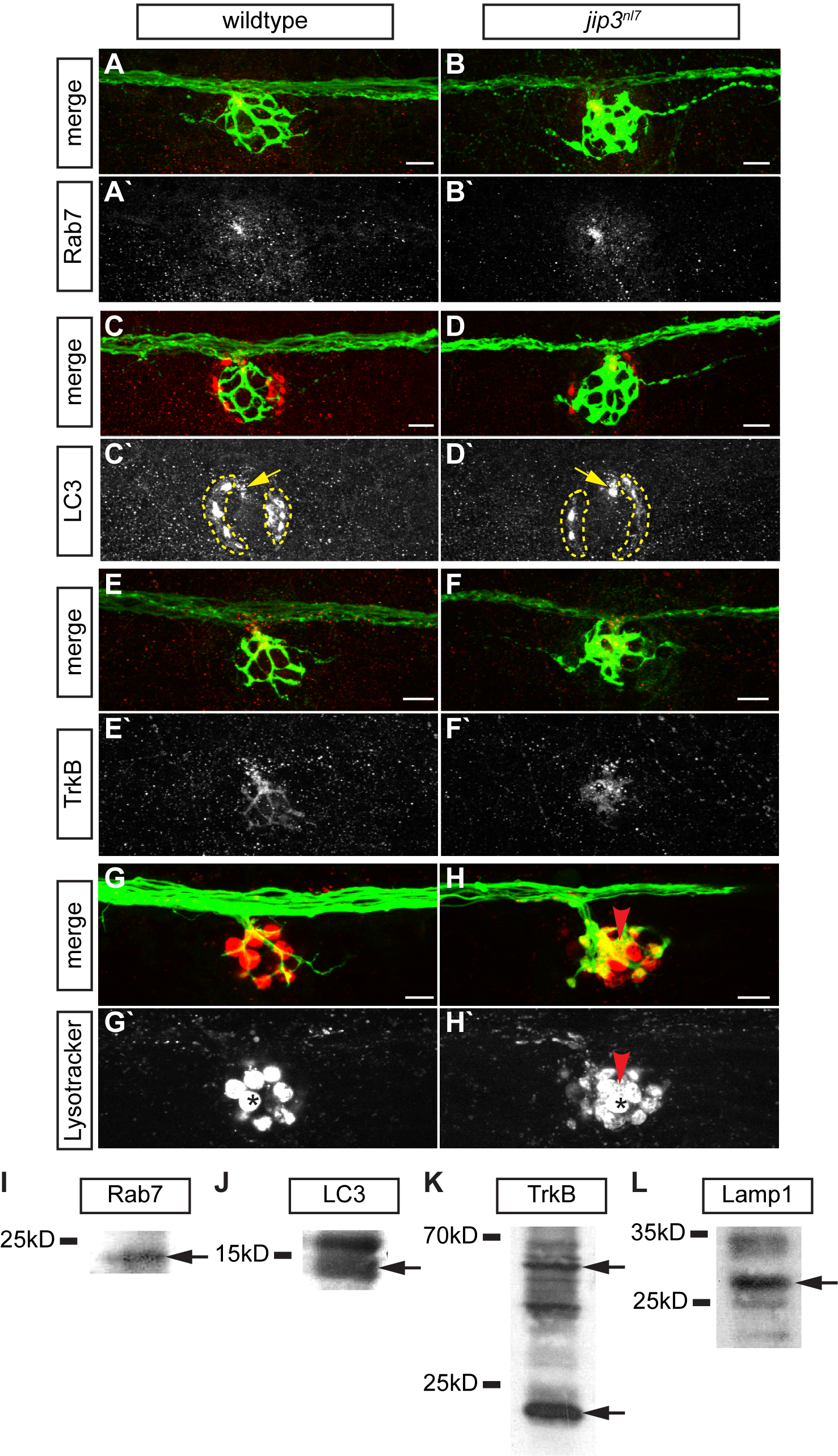

Supplement: Figure S3 — Lysosomes, but not late endosomes, signaling endosomes or autophagosomes, accumulated in jip3nl7 axon terminals. (A–H) The density of late endosomes, autophagosomes, TrkB, and lysosomes were assayed in wildtype and jip3nl7 axon terminals by immunolabeling (A–F) and live imaging (G,H). (A,B) Rab7, a marker of late endosomes, was unchanged in jip3nl7 axon terminals. (C,D) LC3, a marker of autophagosomes, was unchanged in jip3nl7 axon terminals (yellow arrow). Note the high LC3 expression in NM support cells (dotted outline). (E,F) TrkB levels were slightly decreased in jip3nl7 axon terminals. (G,H) Lysotracker red staining at 5 dpf revealed elevated levels of lysosomes in jip3nl7 axon terminals (red arrowhead). Asterisk points out representative hair cells. This cell type has high Lysotracker labeling due to the large number of acidic vesicles. (I–L) Western blot analyses of 3 dpf embryo lysates demonstrate the specificity of the antibodies used. Arrows indicate bands corresponding to those that match the size of the predicted zebrafish protein orthologs. (TIF) [file pgen.1003303.s003.tif]

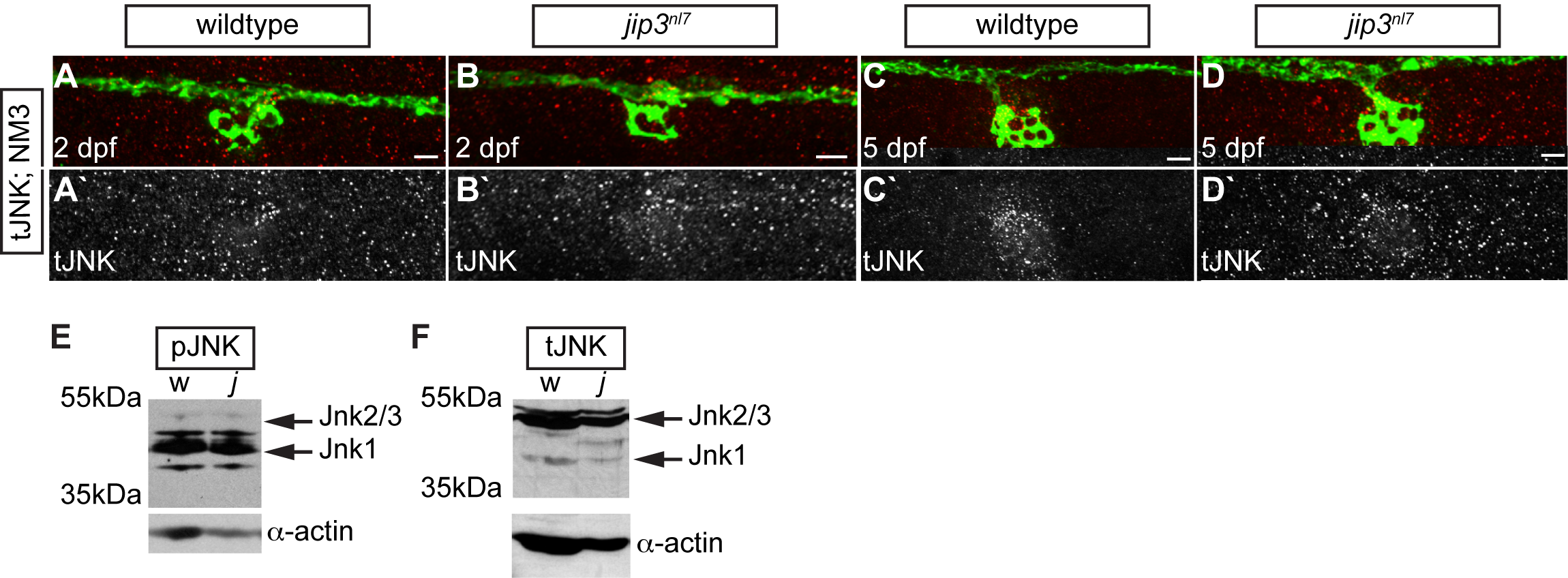

Supplement: Figure S4 — Total JNK levels were not elevated in jip3nl7. (A–D) Total JNK (tJNK) levels were unchanged in jip3nl7axon terminals at 2 and 5 dpf. (E,F) Western blot analysis of 3 dpf whole embryo extracts indicated overall levels of pJNK (E) and tJNK (F) were not changed in jip3nl7 (j) compared to wildtype (w). α-actin control below. Scale bars = 10 µm. (TIF) [file pgen.1003303.s004.tif]

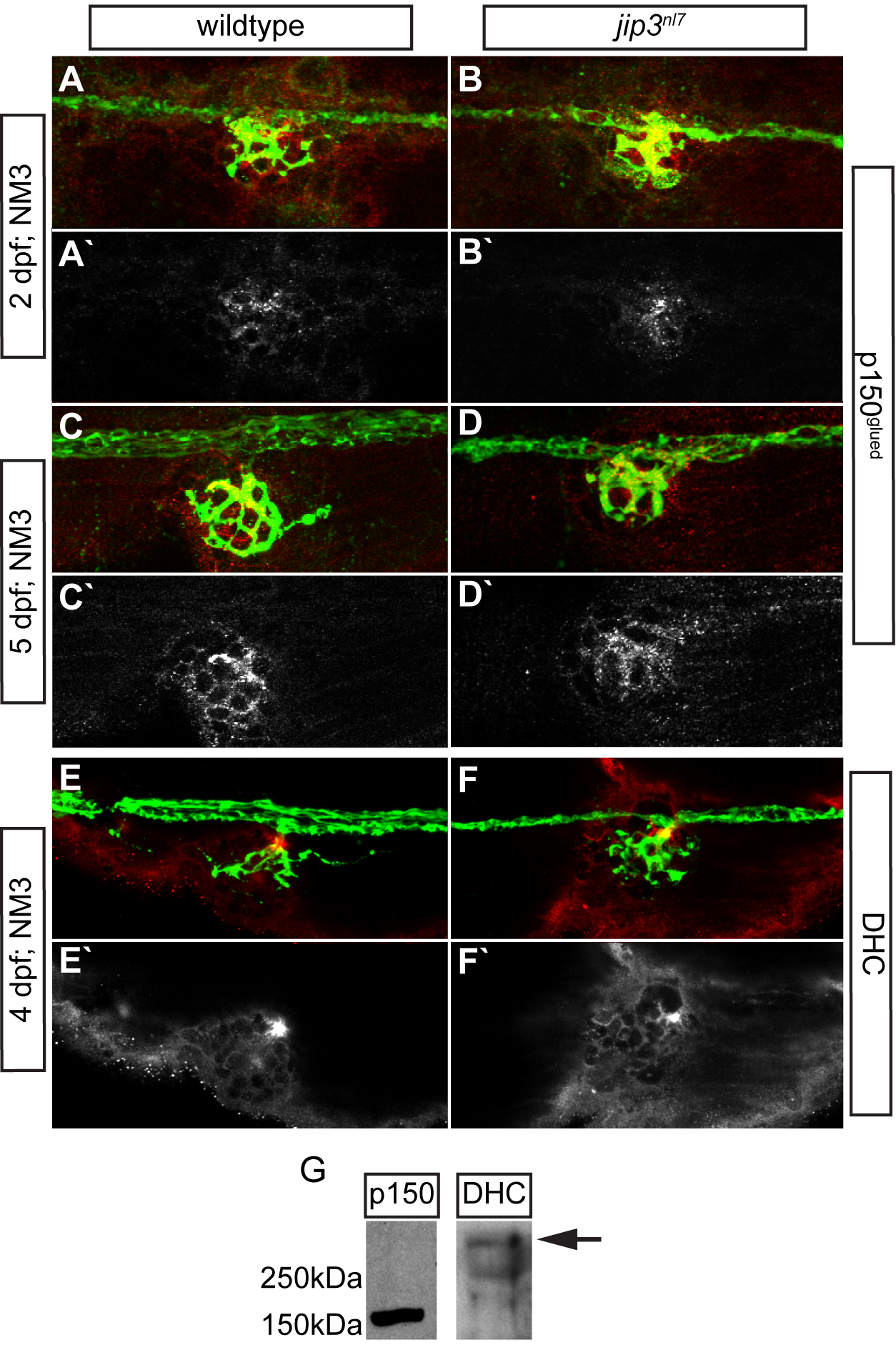

Supplement: Figure S5 — Components of the dynein retrograde motor complex were present at jip3nl7 axon terminals. (A–E) Immunolabeling for two components of the dynein motor complex, p150glued (A–D; 2 and 5 dpf) and dynein heavy chain (E,F; 4 dpf) demonstrated that levels and distribution of these dynein motor components in axon terminals were similar between jip3nl7 and wildtype controls. neurod:EGFP transgene carriers were used to label the pLL nerve (green). The middle portion of the pLL nerve at NM3 is shown in A–F. (G) Western blot analysis of 4 dpf whole embryo extracts demonstrated the specificity of the respective antibodies (arrow indicates DHC band based on predicted molecular weight). (TIF) [file pgen.1003303.s005.tif]

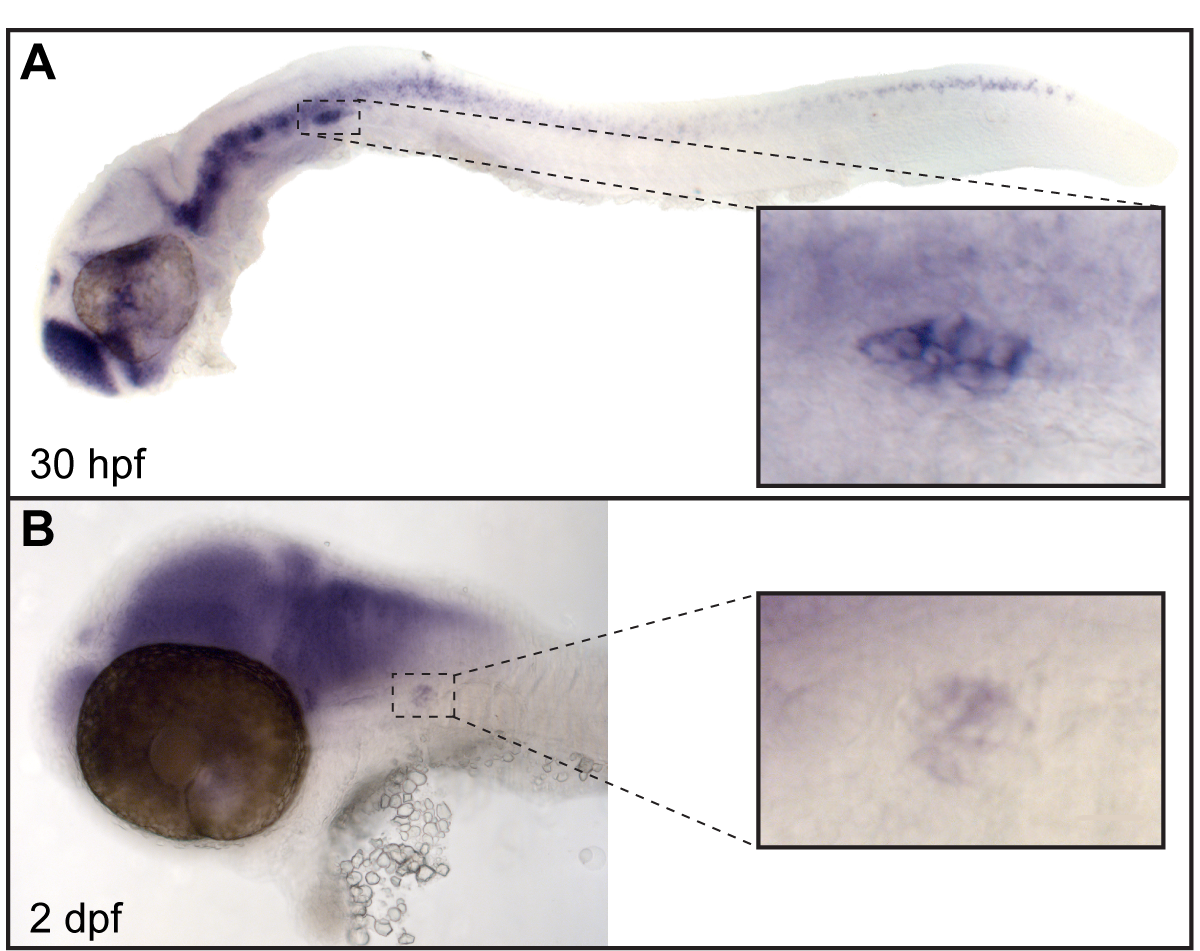

Supplement: Figure S6 — jnk3 is expressed in the peripheral and central nervous systems of the zebrafish embryo. (A) In situ hybridization for jnk3 at 30 hpf revealed expression in the central and peripheral nervous system, including the pLL ganglion (inset). (B) jnk3 expression in these regions, including the pLL ganglion (inset), persisted at 2 dpf. (TIF) [file pgen.1003303.s006.tif]

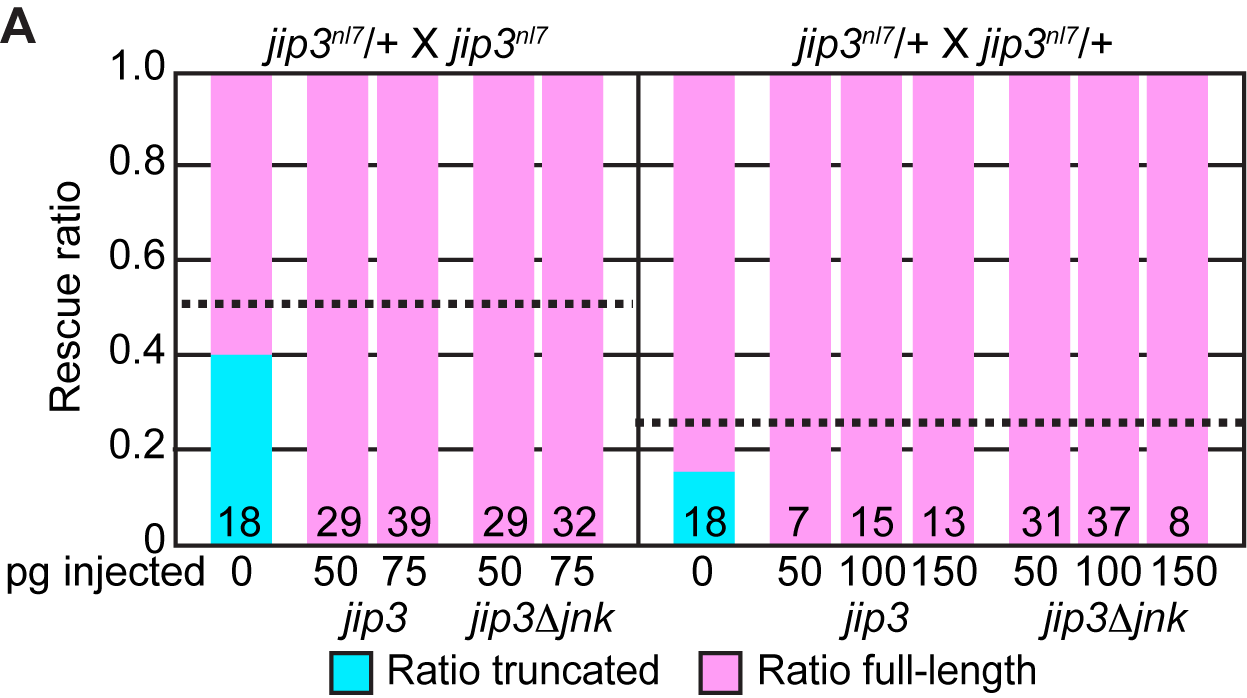

Supplement: Figure S7 — Jip3-JNK interaction did not regulate axon extension. Injection of mRNA encoding both full-length Jip3 and Jip3 lacking the JNK binding domain (Jip3ΔJNK) rescued axon length in jip3nl7. Fraction of embryos expected to have truncated nerves in the respective crosses is indicated by the dashed line (50% in the homozygous X heterozygous cross and 25% in the heterozygous incross). Number of embryos analyzed and amount of DNA injected is indicated for each bar. (TIF) [file pgen.1003303.s007.tif]
